# Overweight, obesity, and individual symptoms of depression: A multicohort study with replication in UK Biobank

**DOI:** 10.1016/j.bbi.2022.07.009

**Published:** 2022-10

**Authors:** Philipp Frank, Markus Jokela, G. David Batty, Camille Lassale, Andrew Steptoe, Mika Kivimäki

**Affiliations:** a Research Department of Epidemiology and Public Health, University College London, 1-19 Torrington Place, WC1E 6BT London, UK; b Department of Psychology and Logopedics, Faculty of Medicine, University of Helsinki, Haartmaninkatu 3, Helsinki 00290, Finland; c Hospital del Mar Research Institute (IMIM), Dr Aiguader 88, 08003 Barcelona, Spain; d Research Department of Behavioural Science and Health, University College, London, 1-19 Torrington Place, WC1E 7HB London, UK; e Clinicum, Faculty of Medicine, University of Helsinki, Tukholmankatu 8 B, FI-00014 Helsinki, Finland

**Keywords:** Overweight, Obesity, Depression, Symptoms of depression, Multicohort study, UK Biobank

## Abstract

**Objectives:**

Obesity is associated with increased risk of depression, but the extent to which this association is symptom-specific is unknown. We examined the associations of overweight and obesity with individual depressive symptoms.

**Methods:**

We pooled data from 15 population-based cohorts comprising 57,532 individuals aged 18 to 100 years at study entry. Primary analyses were replicated in an independent cohort, the UK Biobank study (n = 122,341, age range 38 to 72). Height and weight were assessed at baseline and body mass index (BMI) was computed. Using validated self-report measures, 24 depressive symptoms were ascertained once in 16 cross-sectional, and twice in 7 prospective cohort studies (mean follow-up 3.2 years).

**Results:**

In the pooled analysis of the primary cohorts, 22,045 (38.3 %) participants were overweight (BMI between 25 and 29.9 kg/m^2^), 12,025 (20.9 %) class I obese (BMI between 30 and 34.9 kg/m^2^), 7,467 (13.0 %) class II-III obese (BMI ≥ 35 kg/m^2^); and 7,046 (12.3 %) were classified as depressed. After multivariable adjustment, obesity class I was cross-sectionally associated with 1.11-fold (95 % confidence interval 1.01–1.22), and obesity class II-III with 1.31-fold (1.16–1.49) higher odds of overall depression. In symptom-specific analyses, robust associations were apparent for 4 of the 24 depressive symptoms (‘could not get going/lack of energy’, ‘little interest in doing things’, ‘feeling bad about yourself, and ‘feeling depressed’), with confounder-adjusted odds ratios of having 3 or 4 of these symptoms being 1.32 (1.10–1.57) for individuals with obesity class I, and 1.70 (1.34–2.14) for those with obesity class II-III. Elevated C-reactive protein and 21 obesity-related diseases explained 23 %-31 % of these associations. Symptom-specific associations were confirmed in longitudinal analyses where obesity preceded symptom onset, were stronger in women compared with men, and were replicated in UK Biobank.

**Conclusions:**

Obesity is associated with a distinct set of depressive symptoms. These associations are partially explained by systemic inflammation and obesity-related morbidity. Awareness of this obesity-related symptom profile and its underlying biological correlates may inform better targeted treatments for comorbid obesity and depression.

## Highlights

• Whether the association between excess body weight and depression is attributable to specific depressive symptoms rather than overall depression remains uncertain.
• Obesity was associated with a distinct set of depressive symptoms characterised by low mood, little interest in doing things, feelings of inadequacy, and lack of energy.
• The associations of obesity with other depressive symptoms and overall depression were weaker.
• Systemic inflammation and obesity-related morbidity may be important mechanisms.

## 1 Introduction

The global prevalence of obesity and obesity-related disease burden has increased markedly over recent decades (Dai et al., 2020, Vos et al., 2020). Concomitantly, mental health problems are a major contributor to disease burden in high-income countries, with depression and other psychiatric disorders ranking among the top ten causes of years lived with disability (Reiner et al., 2019). Worldwide, over 650 million people live with obesity (World Health Organization, 2021), and >260 million are affected by depression (Vos et al., 2017). Obesity and depression are both independently linked to an elevated risk of an array of morbidity (Harshfield et al., 2020, Kivimäki et al., 2017) and mortality (Cuijpers et al., 2014, Di Angelantonio et al., 2016) outcomes. Current epidemiological evidence also suggests that these two conditions are often comorbid, and that their relationship is likely to be bidirectional, with stronger associations evident in cases of severe obesity and in women compared with men (Luppino et al., 2010, Mannan et al., 2016, Milaneschi et al., 2019, Vancampfort et al., 2013).

Depression diagnosis captures a heterogeneous group of patients with varying types of symptom expressions (Milaneschi et al., 2020, Silva et al., 2020), and this may complicate research, clinical decision-making, and treatment (Milaneschi et al., 2020). Therefore, dissecting depression heterogeneity by ascertaining symptom-specific associations with excess body weight may provide novel insights into the obesity-depression link. To date, however, studies on obesity and depression have almost exclusively focused on broader diagnostic categories as opposed to individual symptoms of depression (Badini et al., 2020, Brailean et al., 2020, Milaneschi et al., 2017b); are characterized by smaller sample sizes (Alshehri et al., 2019, Badini et al., 2020, Milaneschi et al., 2017b); failed to disaggregate obesity into its clinically relevant obesity classes, which may have masked important differences by obesity subgroups (Alshehri et al., 2019, Badini et al., 2020, Kappelmann et al., 2021, Milaneschi et al., 2017b); and utilized cross-sectional designs that do not have the capacity to examine the temporal sequence between obesity and depressive symptoms (Alshehri et al., 2019, Brailean et al., 2020). In addition, most research in this field has not included subgroup analyses to test the robustness of associations across multiple cohorts from different countries (Alshehri et al., 2019, Badini et al., 2020, Brailean et al., 2020, Kappelmann et al., 2021, Milaneschi et al., 2017b), nor explored potential mediating factors such as systemic inflammation and obesity-related medical diseases to understand underlying mechanisms (Alshehri et al., 2019, Brailean et al., 2020). While some smaller-scale investigations have suggested that obesity is primarily associated with an atypical symptom profile of depression (e.g., mood reactivity, leaden paralysis, increased appetite or weight gain, interpersonal rejection sensitivity, and hypersomnia) (Milaneschi et al., 2020, Silva et al., 2020), this requires confirmation in better-powered studies that offer a broad spectrum of depressive symptoms.

To address these limitations, we conducted a large multicohort study of 15 population-based cohorts with replication in UK Biobank to investigate the cross-sectional and longitudinal associations of overweight, obesity class I, and obesity class II-III with overall depression status and 24 individual depressive symptoms. In addition, we estimated the extent to which symptom-specific associations were attributable to systemic inflammation and obesity-related morbidity.

## 2 Methods

### 2.1 Study population

To identify studies with individual-level data for this pooled multicohort analysis, we systematically searched the collections of the UK Data Service (https://ukdataservice.ac.uk, a total of 8030 studies), the Inter-University Consortium for Political and Social Research (https://www.icpsr.umich.edu/icpsrweb/ICPSR/, 15 366 studies), and the Individual-Participant Data meta-analysis in Working Populations (IPD-Work) consortium (12 studies) (Kivimäki et al., 2012). We identified 15 independent population-based cohort studies, initiated between 1985 and 2018, with relevant data on the exposure, outcome, and covariates (appendix, p 6). These were from the UK (Whitehall II, the English Longitudinal Study of Ageing [ELSA], Understanding Society [UKHLS]), Ireland (the Irish Longitudinal Study on Ageing [TILDA]), the USA (the National Health and Nutrition Examination Survey [NHANES], Midlife in the United States [MIDUS], the National Social Life, Health, and Aging Project [NSHAP], Health and Retirement Study [HRS]), Mexico (the Mexican Health and Ageing Study [MHAS]), Costa Rica (the Costa Rican Longevity and Healthy Ageing Study [CRELES]), and Taiwan (the Social and Biomarkers of Ageing Study [SEBAS]). Individuals under age 18 and those with missing data on the outcome, exposure, and/or covariates were excluded from the present analyses. To examine the robustness of our results in an independent population, we repeated cross-sectional analyses in 122,341 participants from the UK Biobank study.

Ethical approval for individual studies was granted by the relevant local or institutional ethical review panels. Written informed consent was provided prior to participation in all studies.

### 2.2 Measures

#### 2.2.1 Assessment of body mass index and covariates

Body mass index (BMI) was computed from weight in kilograms divided by height in meters squared (kg/m^2^). We divided continuous BMI scores into four categories: normal weight (≥18.5 kg/m^2^ to < 25 kg/m^2^), overweight (≥25 kg/m^2^ to < 30 kg/^2^), class I obesity (≥30 kg/m^2^ to < 35 kg/m^2^), and class II–III obesity (≥35 kg/m^2^) (World Health Organization, 2000).

Socio-demographic variables were sex and age. As previously (Frank et al., 2021), educational qualification (low, medium, high) was used as an indicator of socio-economic position. Lifestyle factors included self-reported smoking status (yes, no), alcohol consumption (none/low, medium, high), and physical activity (physically active, not active). Chronic illness covariates comprised self-reported coronary heart disease, stroke, diabetes, and cancer. In UK Biobank, vascular/heart diseases were measured via self-reported doctor diagnosed indications of any of the following conditions: heart attack, angina, stroke, and high blood pressure.

In all studies, we used serum or plasma levels of C-reactive protein (CRP) as a marker of systemic inflammation (mg/L). Obesity-related morbidity, measured in UK Biobank, included 21 non-overlapping conditions covering endocrine (all adult-onset diabetes), cardiovascular (hypertension, angina, myocardial infarction, heart failure, arrhythmia, cerebral infarction, deep vein thrombosis, pulmonary embolism), digestive (pancreatitis, liver disease), infectious (bacterial infections), musculoskeletal (gout, osteoarthritis, back pain), respiratory (asthma), malignant (kidney cancer), skin (skin infections and eczema), blood (anemia), genitourinary (renal failure), and nervous system (sleep disorders) diseases. These conditions were ascertained from linkage data to the UK National Health Service (NHS) Hospital Episode Statistics database for hospital admissions (Kivimäki et al., 2022), and were coded according to the International Classification of Diseases 10th Revision (ICD-10).

#### 2.2.2 Assessment of depressive symptoms

Data on overall depression status (i.e., elevated versus non-elevated levels of depressive symptoms) and a total of 24 individual symptoms of depression were drawn from validated self-report measures for depressive symptoms (appendix, p 8). Table 1 provides the full list of symptoms included in the analyses. Response scales varied by measure and study and were harmonized by coding items as presence (1) versus absence (0) of the symptom. In seven cohorts, 21 symptoms of depression were measured repeatedly, at baseline when BMI was assessed, and 1 to 5 years later. For eight cohorts, we had follow-up data on BMI to assess whether depressive symptoms preceded weight gain. Further details on measure-specific response scales and harmonized cut-off values are available in the appendix, p 8.

**Table 1:** Depressive symptoms under study.

		**Multicohort data**			**UK Biobank**	
**Symptom domain**	**Depressive symptom**	**Prevalence (%)**	**N (total)**	**N (cohorts)**	**Prevalence (%)**	**N (total)**
Emotional symptoms	Felt hopeless about the future	18.1	10931	4	–	–
	Felt sad	14.1	26545	7	–	–
	Felt unhappy	12.5	34406	9	–	–
	Felt lonely	11.5	27134	8	–	–
	Did not enjoy life	10.8	34406	9	–	–
	Felt depressed	10.5	55774	13	3.23	122341
	Bothered by things	6.6	9762	3	–	–
	Felt fearful	3.8	9762	3	–	–
	Life had been a failure	3.5	9762	3	–	–
Physical symptoms	Sleep was restless	21.4	56363	14	14.56	122341
	Could not get going/energy	19.7	50840	14	10.55	122341
	Felt everything was an effort	15.9	27134	8	–	–
	Changes in appetite	7.4	34687	10	5.05	122341
	Talked less than usual	6.9	9762	3	–	–
	Moving or speaking slowly/too fast	3.7	22537	5	1.29	122341
	Had crying spells	2.5	9762	3	–	–
Cognitive symptoms	Little interest in doing things/ unmotivated	11.3	31257	6	3.69	122341
	Difficulties concentrating	9.0	38991	9	3.52	122341
	People were unfriendly	3.1	12150	5	–	–
Perception of self	Felt worse than others	13.6	9762	3	–	–
	Feeling bad about yourself	5.3	22537	5	4.04	122341
	Could not shake off the blues	4.3	9762	3	–	–
	People dislike me	2.4	11561	4	–	–
Self-harm symptom	Thought you would be better off dead	1.1	22537	5	0.78	122341

*Note*: Data for all symptoms were not available in the UK Biobank study.

### 2.3 Statistical analysis

We performed a two-step individual-participant-data *meta*-analysis. First, we conducted multiple logistic regression analyses to compute odds ratio (OR) with 95 % accompanying confidence intervals (CIs) separately in each cohort. Second, study-specific point estimates and standard errors were aggregated using random-effects *meta*-analysis. In comparison to fixed-effects, random-effects models typically provide a more conservative estimate (Higgins et al., 2003). We examined heterogeneity in study-specific point estimates by computing *I ^2^* and *τ ^2^* statistics. *I ^2^* describes the total proportion of variation in effect sizes that is not due to sampling error. *τ ^2^* indicates inter-cohort variance.

In addition to minimally adjusted effect estimates (age and sex), we generated a series of multivariable models adjusting effect estimates for (i) age, sex, and socio-economic position; (ii) age, sex, and behavioral factors; (iii) age, sex, socio-economic position, and behavioral factors (appendix, pp 11–13); (iv) age, sex, and illness-related factors; and (v) age, sex, illness-related factors, and systemic inflammation. CRP values were log-transformed because of their skewed distribution.

#### 2.3.1 Sensitivity analyses

We also conducted a series of sensitivity analyses. First, to assess potential differences in symptom-specific associations between cohorts that computed BMI on the basis of self-reported rather than nurse-measured height and weight, we conducted an additional analysis stratifying cohorts by BMI ascertainment. Second, to examine whether excess body weight preceded or followed individual symptoms of depression, analyses were repeated longitudinally, additionally adjusting effect estimates for the respective depressive symptom or BMI category at baseline. In addition, we repeated symptom-specific longitudinal analyses excluding depressed individuals at baseline to control for potential use of anti-depressant medication. Third, to assess whether the identified obesity-related symptoms represented a distinct depression profile, we investigated the odds for overweight, class I-, and class II-III obese individuals to experience one (versus zero) symptom, two (versus zero or one) symptoms, and three or more (versus zero, one or two) symptoms. Fourth, to examine the robustness of obesity-symptom associations in an external population, cross-sectional analyses were repeated in UK Biobank. Fifth, using UK Biobank data, we examined the extent to which these associations were explained by systemic inflammation (CRP) and the presence/history of one or more of 21 obesity-related diseases. To this end, we calculated the percentage of attenuation following adjustment for CRP and a history of or current obesity-related diseases [1 = yes, 0 = no] using the following formula: (β [base adjusted] – β [base and confounder adjusted]) / (β [base adjusted]) × 100, with β being the log_e_-transformed OR point estimate.

The strength of evidence for each BMI–depressive symptom association was evaluated based on the following criteria (Siontis & Ioannidis, 2011): a ‘large’ magnitude of the effect was denoted by an OR in the basic model ≥ 1.20 and statistical significance at a Bonferroni corrected alpha-level, P < 4.17x10^-4^ (i.e., adjustment for 120 tests); ‘moderate’ by an OR between 1.10 and 1.19 and P < 4.17x10^-4^; and ‘small’ by an OR < 1.10, but P < 4.17x10^-4^; robustness to multivariable adjustments; temporality (‘yes’, a significant association in the longitudinal analysis; otherwise ‘no’); heterogeneity in study-specific estimates (‘low’, I^2^ < 25 %; ‘moderate’, I^2^ between 25 % and 50 %; ‘high’, I^2^ > 50 %); generalizability across subgroups (men, women, age groups 18 to 60 and > 60 years, and a subgroup of people with depression); and replication of symptom-specific associations in an independent population from the UK Biobank (‘yes’, a significant cross-sectional association; otherwise ‘no’).

All study-specific analyses were conducted in Stata version 16.0, whereas random-effects *meta*-analyses were performed using the ‘*metafor*’ (Viechtbauer, 2010) package in RStudio version 1.4.1106. Statistical code is provided in Web appendix 3 in the supplement.

## 3 Results

### 3.1 Random-effects *meta*-analyses of the primary cohorts

The primary analysis was based on pooled data from 15 cohort studies comprising 57,532 individuals (29,890 women) with a mean age of 57.5 (SD = 16.0) years. A total of 12.3 % of participants were classified as depressed (62.9 % were women). The prevalence of depressive symptoms across cohorts varied between 1.1 % *(‘suicidal ideation’*) and 21.4 % (‘*sleep problems’*) (Table 1). The proportion of participants with healthy weight was 27.8 %, whereas 38.3 % were overweight, 20.9 % obese class I, and 13.0 % obese class II-III (appendix, p 7).

While there was no evidence for a cross-sectional association between overweight and overall depression status (age- and sex-adjusted odds ratio [OR] 0.95; 95 % CI, 0.87 to 1.03), the odds ratio was 1.29 (95 % CI, 1.18 to 1.41) for obesity class I and 1.69 (95 % CI, 1.49 to 1.91) for obesity class II-III (appendix, p 10). After adjustment for demographic characteristics, chronic illnesses, and CRP, obesity class I was associated with a 1.11-fold (95 % 1.01 to 1.22), and obesity class II-III with a 1.31-fold (95 % 1.16 to 1.49) increased risk of depression. In multivariable-adjusted symptom-specific analyses, and after Bonferroni multiple testing correction, we observed robust associations for six of the 24 depressive symptoms (appendix, p 10). Compared with participants in the normal weight range, those with obesity class I and/or obesity class II-III, but not overweight, had significantly higher odds of reporting the following symptoms: ‘*could not get going / lack of energy’, ‘felt everything was an effort’, ‘little interest in doing things’, ‘felt bad about myself’, ‘felt depressed’*, and ‘*could not shake off the blues’*. This pattern of results was also noted in a sensitivity analysis focusing exclusively on depressed individuals, although the magnitude of these relationships was slightly attenuated and less precisely estimated (appendix, p 16).

**Fig. 1:**
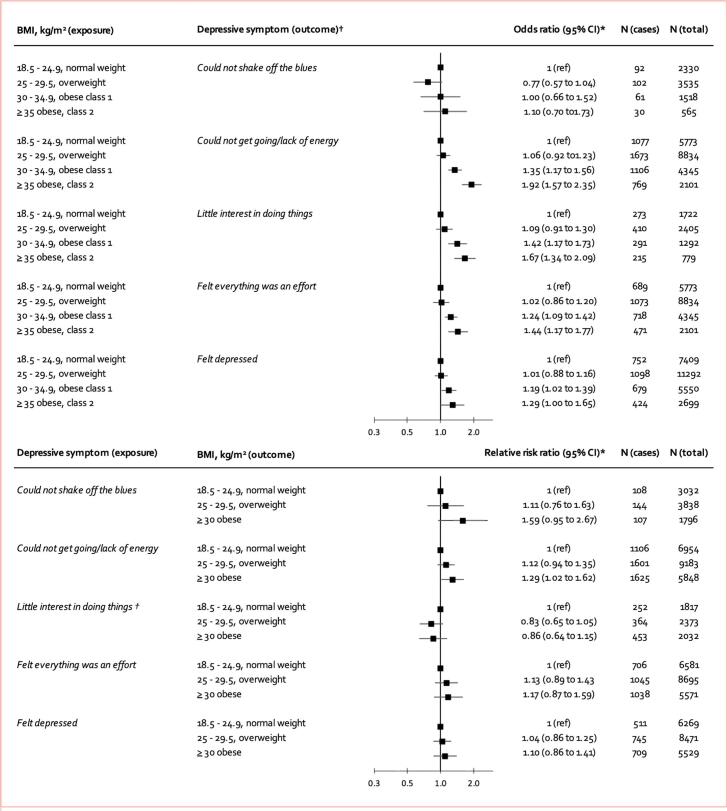
*Adjusted for age, sex, and baseline depressive symptom/BMI category. †No longitudinal data were available for symptom 'felt bad about myself'. In subsequent analyses, we did not consider the symptoms ‘could not shake off the blues’ because of no support for a longitudinal association, and 'felt everything was an effort' because no data were available in the replication cohort. Note: Analyses of the association between depressive symptoms (exposure) and BMI (outcome) are based on multinomial regression analysis.

We examined the temporal order between BMI and five of the six symptoms that were robust to multivariable adjustment in cross-sectional analyses (there were no longitudinal data available for the symptom *‘felt bad about myself’*)*.* Except for ‘*could not shake off the blues’*, longitudinal associations indicated that obesity preceded the risk of experiencing the four remaining symptoms 1 to 5 years later (Fig. 1). For one symptom (*‘could not get going / lack of energy*’) there was additional evidence for a bidirectional relationship. Sensitivity analyses excluding participants with the respective depressive symptom at baseline confirmed symptom-onset (appendix, p 22). Exclusion of people with baseline (overall) depression did not markedly affect the results (appendix, p 17). As obesity did not predict the symptom ‘*could not shake off the blues’,* and because there were no data available for the symptom *‘everything was an effort’* in the replication cohort (Table 1), we did not consider these two symptoms in further analyses.

Heterogeneity in study-specific effect estimates, indexed by *I^2^* and *τ^2^*, varied by cohort, obesity category, and symptom, and was low to high for ‘*could not get going/lack of energy’* and *‘little interest in doing things*’ and small for *‘felt bad about myself’* and *‘felt depressed’* (appendix, pp 26–34). In addition, the sensitivity analysis stratifying cohorts by BMI ascertainment (i.e., self-reported versus nurse/staff-measured) revealed no significant differences by type of measurement (appendix, p 23). Thus, all further analyses were focused on these four symptoms.

Subgroup analyses stratified by age and sex for the four obesity-related symptoms can be found in the supplement (appendix, p 15). Overlapping point estimates and 95 % CIs indicated that there were no differences between younger and older adults in the primary analysis. In contrast, we found evidence for differential associations in sex-stratified analyses, such that, compared with men, excess body weight in women was associated with higher odds of the symptoms *‘could not get going/lack of energy’*, *‘little interest in doing things’*, *‘felt bad about myself’* and *‘felt depressed’*. In men, most associations were weaker but statistically significant at conventional levels.

**Fig. 2:**
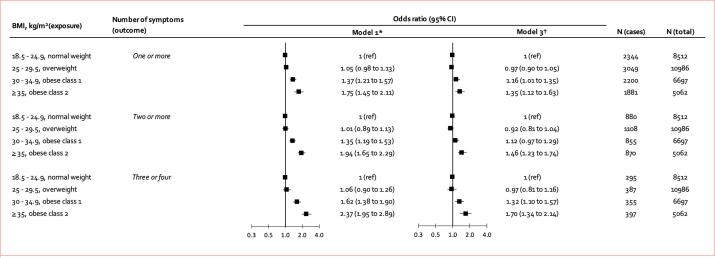
Minimally and multivariable adjusted cross-sectional associations of body mass index with experiencing 1, 2, or 3–4 obesity-related symptoms of depression (random-effects *meta*-analysis of cohort studies) *Adjusted for age and sex. †Adjusted for age, sex, prevalent or history of coronary heart disease, stroke, diabetes, cancer, and C-reactive protein level at baseline.

In Fig. 2, we show the cross-sectional odds ratios for individuals with overweight and obesity to experience one (versus zero), two (versus zero or one), or three to four (versus zero to two) of the identified obesity-related depression symptoms (i.e., ‘*could not get going/lack of energy’, ‘little interest in doing things’, ‘felt bad about myself’* and *‘felt depressed’*). After adjustment for age and sex, obesity class I and obesity class II-III were associated with 1.62-fold (1.38 to 1.90) and 2.37-fold (1.95 to 2.89) increased odds of reporting three or four of the obesity-related symptoms, respectively. After further adjustment for socio-demographic characteristics, chronic illnesses, and CRP, these associations were slightly weaker but remained statistically significant at conventional levels (obesity class I: 1.32; 95 %, 1.10 to 1.57: obesity class II-III:1.70; 95 %,1.34 to 2.14).

### 3.2 Replication in UK Biobank

**Fig. 3:**
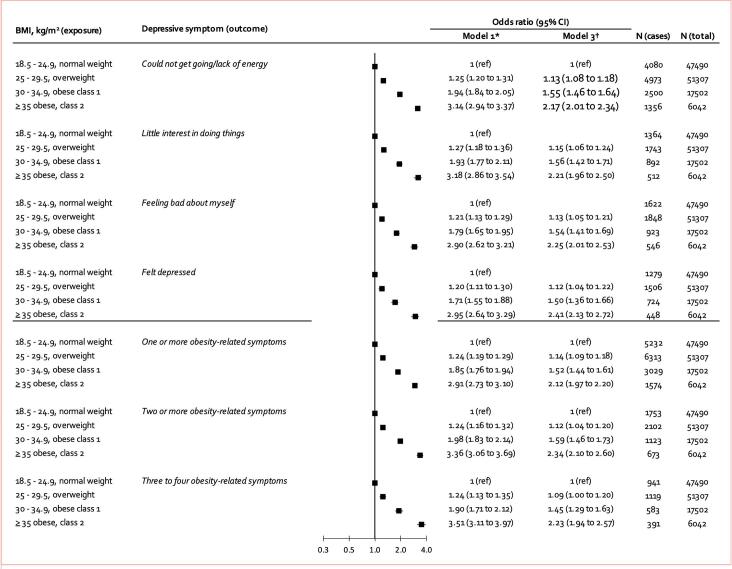
Minimally and multivariable adjusted cross-sectional associations of body mass index with 4 individual symptoms of depression and experiencing 1, 2, or 3–4 obesity-related symptoms of depression (UK Biobank) *Adjusted for age and sex. †Adjusted for age, sex, prevalent or history of coronary heart disease, stroke, diabetes, cancer, and C-reactive protein level at baseline.

The replication cohort consisted of 122,341 UK Biobank participants (66,520 women, mean age 55.8 years, SD = 7.8). Like in the pooled analysis of the primary cohorts, symptom-specific prevalence was lowest for ‘*suicidal ideation’* (0.8 %) and highest for ‘*sleep problems’* (14.6 %). While 38.8 % of UK Biobank participants fell within the normal weight range, 41.2 % were classified as overweight, 14.3 % as obese class I, and 4.9 % as obese classes II-III (appendix, p 7). Replication analysis confirmed dose–response associations between BMI and ‘*could not get going / lack of energy’, ‘little interest in doing things’, ‘felt bad about myself’*, and *‘felt depressed’,* with confounder-adjusted odds ratio of having 3 or 4 of these symptoms being 1.45 (1.29–1.63) for individuals with obesity class I, and 2.23 (1.94–2.57) for those with obesity class II-III (Fig. 3). Replication of longitudinal analyses was not possible due to the lack of follow-up data on depressive symptoms in UK Biobank.

Lastly, in analyses examining the role of systemic inflammation and obesity-related diseases in the four robust obesity-symptom associations [UK Biobank], the results revealed that approximately-one third (23.5 % to 31.2 %) of these associations was attributable to elevated levels of CRP and a history of or current obesity-related morbidity (Table 2).

**Table 2:** The contribution of systemic inflammation and obesity-related morbidity to the associations of obesity with 4 obesity-related depressive symptoms in UK Biobank.

**BMI, kg/m2 (exposure)**	**Depressive symptom (outcome)**	**Odds ratio (95 % CI)**							**N (cases)**	**N (total)**
		Model 1*	**Model 2†**	**Attenuation (%)**	**Model 3††**	**Attenuation (%)**	**Model 4‡**	**Attenuation (%)**		
18.5–24.9, normal weight	*Could not get going/lack of energy*	1 (ref)	1 (ref)		1 (ref)		1 (ref)		4804	55,093
≥ 30, obese		2.27 (2.17 to 2.37)	1.89 (1.80 to 1.99)	22.35	2.09 (2.00 to 2.19)	10.08	1.77 (1.69 to 1.86)	30.35	4723	28,292
18.5–24.9, normal weight	*Little interest in doing things*	1 (ref)	1 (ref)		1 (ref)		1 (ref)		1617	55,093
≥ 30, obese		2.23 (2.08 to 2.39)	1.87 (1.73 to 2.03)	21.95	2.09 (1.94 to 2.24)	8.08	1.77 (1.64 to 1.92)	28.81	1697	28,292
18.5–24.9, normal weight	*Feeling bad about myself*	1 (ref)	1 (ref)		1 (ref)		1 (ref)		1897	55,093
≥ 30, obese		2.11 (1.97 to 2.25)	1.86 (1.73 to 2.00)	16.89	1.99 (1.86 to 2.13)	7.84	1.77 (1.65 to 1.91)	23.53	1794	28,292
18.5–24.9, normal weight	*Felt depressed*	1 (ref)	1 (ref)		1 (ref)		1 (ref)		1511	55,093
≥ 30, obese		2.07 (1.92 to 2.23)	1.74 (1.60 to 1.89)	23.87	1.94 (1.80 to 2.09)	8.91	1.65 (1.52 to 1.79)	31.17	1453	28,292

*Adjusted for age and sex.

†Adjusted for age, sex, and C-reactive protein level at baseline.

††Adjusted for age, sex, and history of/current at least one of 21 common obesity-related diseases at baseline.

‡ Adjusted for age, sex, C-reactive protein, and current or a history of at least one of 21 common obesity-related diseases at baseline.

## 4 Discussion

In this pooled analysis of 15 population-based cohort studies with replication in UK Biobank, our main finding was that obesity, but not overweight, was robustly associated with 4 of the 24 depression-related symptoms included in this study. These symptoms covered physical (‘could not get going/lack of energy’), cognitive (‘little interest in doing things’), emotional (‘felt depressed’), and self-perception (‘felt bad about myself’) domains and followed a dose–response pattern, with consistently stronger effect estimates evident in cases of severe obesity (BMI ≥ 35). The identified associations were not attributable to socio-demographic, lifestyle, or illness-related factors, including systemic inflammation and 21 obesity-related diseases. Longitudinal data confirmed temporality with obesity preceding rather than following the development of these symptoms. Stratified analyses revealed no age interaction, suggesting that these results also apply to later-onset symptoms. While evident in both sexes, the associations of obesity with both depressive symptoms and overall depression were stronger in women. Furthermore, the risk for individuals with obesity to experience three or more of the identified symptoms was twice as high as the risk of experiencing depression more generally, supporting a distinct obesity-related symptom profile.

The set of obesity-related depressive symptoms identified in this study is not attributable to any existing diagnostic symptom profiles. We identified two obesity-related symptoms – ‘felt depressed’ and ‘little interest in doing things’ – which correspond to the core features of major depressive disorder; that is, depressed mood and anhedonia (loss of interest or pleasure) (American Psychiatric Association, 2013). Anhedonia also represents the cardinal symptom of melancholic depression. Consistent with previous research (Lamers et al., 2018, Milaneschi et al., 2017a), we found that obesity was also strongly associated with the energy-orientated symptom ‘could not get going/lack of energy’, which overlaps with some features of both major depressive disorder and atypical depression, such as fatigue, lower energy levels, and leaden paralysis.

Some of the symptom-specific associations identified in this study are supported by a recent Mendelian randomization study which reported potential causal associations between genetic variants related to increased BMI and anhedonia, tiredness, and feelings of inadequacy (i.e., feeling bad about yourself) (Kappelmann et al., 2021). In contrast to some earlier investigations (Lamers et al., 2018, Milaneschi et al., 2017a), we found no robust association with the symptom ‘changes in appetite’, which might be explained by the diametrically opposite nature of the item, measuring both increase and decrease in appetite. Taken together, both existing and the current findings suggest that individuals with obesity may endorse typical, atypical, and melancholic symptoms, and that obesity, in particular severe obesity (class II-III obesity), is likely to precede, rather than follow, these symptoms.

### 4.1 Plausible mechanisms linking obesity with depressive symptoms

There are many plausible mechanisms for the obesity-depression link. In the present study, adjustment for CRP exerted the greatest attenuating effect (about 20 %) on the identified obesity-depressive symptom associations, supporting a potential mediating role of systemic inflammation. Inflammatory markers such as interleukin-6 (IL-6) and CRP are synthesized by adipocytes (Tilg & Moschen, 2006), and higher levels of body fat, in particular visceral fat, are related to both metabolic inflammation and depression-related symptoms (Milaneschi et al., 2019, Speed et al., 2019). Underlying obesity-induced medical diseases may also contribute to the link between obesity and depressive symptoms because they are often co-morbid with depression (Gold et al., 2020, Kivimäki et al., 2022). Our findings suggest that approximately 10 % of the relationship of obesity with the four identified depressive symptoms is attributable to obesity-related morbidity. There are also several other biological mechanisms that could explain the association between obesity and depression. These include a dysregulated hypothalamic pituitary adrenal (HPA)-axis; neuroendocrine changes of the leptin-melanocortin pathway leading to leptin-resistance (Milaneschi et al., 2020); and overlapping genetic variation (e.g., *OLFM4* and *NEGR1*) (Wray et al., 2018). Furthermore, internalization of negative weight stereotypes due to stigmatization is a possible psychological pathway. This might be particularly important for self-perception-related and emotional symptoms such as ‘feeling bad about yourself’ or ‘feeling depressed’. Furthermore, weight bias internalization has been found to be more frequently experienced by women (Pearl & Puhl, 2018), which might explain some of the sex differences observed in the present study.

### 4.2 Implications

Our findings may have implication for the treatment of comorbid obesity and depression. Previous studies have shown that the proportion of patients with treatment resistant depression is higher (Rizvi et al., 2014), and treatment response to selective serotonin reuptake inhibitors (SSRI), such as escitalopram, poorer (Milaneschi et al., 2020) in overweight and obese individuals. Pharmacological add-on therapies targeting weight loss have been suggested to facilitate better treatment outcomes in these patients. For example, the norepinephrine-dopamine reuptake inhibitor bupropion has been found to affect the leptin-melanocortin pathway by stimulating hypothalamic pro-opiomelanocortin neurons, which, in turn, reduces appetite, elevates energy expenditure, and supports weight loss (Anderson et al., 2002, Milaneschi et al., 2020). Accordingly, escitalopram combined with bupropion has been shown to improve the effects of escitalopram in patients with higher BMI (Jha et al., 2018). Our findings raise the hypothesis that people with a distinct obesity-related symptom profile may be more likely to benefit from intensified weight loss interventions as compared to those without such symptom profiles. Furthermore, consideration of depression heterogeneity in future research may lead to significant advances in understanding poorer treatment responses to anti-depressant therapies in patients with comorbid obesity and depression. We argue that clinical trial stratification should be based on symptom profiles, in addition to weight status, rather than overall depression.

The validity of the present study is strengthened by previous research reporting similar associations between obesity and overall depression. Our primary analysis suggests a 1.29-fold increased age- and sex-adjusted risk for overall depression in people with obesity class I, and a 1.69-fold increased risk in those with obesity class II-III. These estimates are broadly consistent with earlier *meta*-analyses reporting minimally adjusted pooled odds ratios for obesity (≥30 kg/m^2^) ranging between 1.14 and 1.42 (Milaneschi et al., 2019).

## 5 Limitations

The interpretation of our findings requires consideration of various limitations. Causal inference is not possible given that all the studies in the present *meta*-analysis are observational. However, causality has been supported by Mendelian randomization studies that used genetic risk scores of BMI as the predictors of depression (Tyrrell et al., 2019, van den Broek et al., 2018) and depressive symptoms (Kappelmann et al., 2021). BMI ascertainment varied by cohort, with some studies using self-reported rather than nurse-administered measures of height and weight. To address this limitation, we conducted a sensitivity analysis stratifying cohorts according to BMI ascertainment, which revealed no significant differences by method. The assessment of depressive symptoms was based on validated self-report measures but not clinical interviews, which is the gold standard in psychiatric research. Furthermore, no data were available for bipolar depression or the full range of symptoms characterizing atypical or melancholic depression subtypes (e.g., leaden paralysis, increases in appetite, mood reactivity); these limitations warrant further investigation. In addition, we had no data on anti-depressant drug treatment. However, excluding people with baseline depression did not markedly affect our results, suggesting that anti-depressant drug treatment is an unlikely source of major confounding for our results.

In conclusion, this multicohort study with replication in independent population showed an association between obesity and a distinct set of depressive symptoms that was partially attributable to systemic inflammation and obesity-related morbidity. This evidence increases understanding of the obesity-depression link and supports symptom-focused approaches to explore comorbid obesity and depression. A new evidence-based dissection of depression heterogeneity, such as ours, can potentially aid treatment selection and inform the search for more effective interventions.

**Disclosures**

*Contributors*

PF and MK generated the idea for the paper. All authors contributed significantly to the conception, design, and analysis or interpretation of data. PF wrote the first draft of the manuscript and other authors were involved in revising it critically for intellectual context. PF, MJ, and MK had full access to the anonymized individual-participant data from all constituent studies and take responsibility for the integrity of the data and the accuracy of the data analyses. The corresponding author attests that all listed authors meet authorship criteria and that no others meeting the criteria have been omitted. The final submission of this paper was approved by all authors. PF, MJ, and MK have verified the underlying data.

*Data sharing*

Syntax for data analysis is provided in the appendix. Our data protection agreements with the participating cohort studies do not allow us to share individual-level data from these studies to third parties. Pre-existing individual-level data access policies for each of the participating cohort studies specify that research data requests can be submitted to each steering committee; these will be promptly reviewed for confidentiality, data protection issues, or intellectual property restrictions and will not unreasonably be refused. Researchers registered with UK Biobank can apply for access to the database by completing an application. This must include a summary of the research plan, data-fields required, any new data or variables that will be generated, and payment to cover the incremental costs of servicing an application (https://www.ukbiobank.ac.uk/enable-your-research/apply-for-access).

## Declaration of Competing Interest

The authors declare that they have no known competing financial interests or personal relationships that could have appeared to influence the work reported in this paper.
