# Supplementary material for: Overweight, obesity, and individual symptoms of depression: A multicohort study with replication in UK Biobank
Source: Brain Behav Immun. 2022 Oct;105:192–200. doi: 10.1016/j.bbi.2022.07.009 (PMC10499756; doi:10.1016/j.bbi.2022.07.009)
Supplement: Supplementary data 1 [file mmc1.docx]

Data supplement for Frank et al. Overweight, Obesity, and Individual Symptoms of Depression: A Multicohort Study with Replication in UK Biobank

**CONTENTS**

[Appendix 1: Description of cohort studies and supplementary analyses with pooled data 3](#_Toc107663089)

[Figure S1. Selection of studies for individual-participant pooled analysis of the association between excess body weight and individual symptoms of depression & selection of participants in the UK Biobank 6](#_Toc107663090)

[eTable S1. Baseline characteristics of the included cohorts 7](#_Toc107663092)

[eTable S2. Study characteristics and method of outcome (depressive symptoms) ascertainment 8](#_Toc107663093)

[eTable S3. Robust, serially adjusted cross-sectional associations of body mass index with 6 top symptoms of depression (random-effects meta-analysis of cohort studies) 10](#_Toc107663094)

[Figure S2a. Serially adjusted cross-sectional associations of body mass index with emotional symptoms of depression (random-effects meta-analysis) 11](#_Toc107663096)

[Figure S2b. Serially adjusted cross-sectional associations of body mass index with physical symptoms of depression (random-effects meta-analysis) 12](#_Toc107663097)

[Figure S2c. Serially adjusted cross-sectional associations of body mass index with cognitive, self-perception-related, and self-harm-related symptoms of depression (random-effects meta-analysis) 13](#_Toc107663098)

[eTable S4. Cross-sectional associations of body mass index with individual symptoms of depression adjusted for chronic disease and C-reactive protein (random-effects meta-analysis) 14](#_Toc107663099)

[Figure S4. Age- and sex-adjusted cross-sectional associations of body mass index with 6 top symptoms among depressed individuals (random-effects meta-analysis) 16](#_Toc107663100)

[Figure S5. Age- and sex-adjusted longitudinal associations of body mass index with 5 top symptoms and overall depression status after excluding participants with depression at baseline (random-effects meta-analysis) 17](#_Toc107663102)

[eTable S5. Serially adjusted cross-sectional associations of body mass index with 4 symptoms with strong evidence against an association with excess body weight (random-effects meta-analysis) 18](#_Toc107663104)

[Figure S6. Age-adjusted sex-stratified cross-sectional associations of body mass index with 4 symptoms with strong evidence against an association with excess body weight (random-effects meta-analysis) 19](#_Toc107663105)

[Figure S7. Sex-adjusted age-stratified cross-sectional associations of body mass index with 4 symptoms with strong evidence against an association with excess body weight (random-effects meta-analysis) 20](#_Toc107663106)

[Figure S8. Age- and sex-adjusted longitudinal associations of body mass index with symptoms with strong evidence against an association with excess body weight (random-effects meta-analysis) 21](#_Toc107663107)

[Figure S9. Age- and sex-adjusted longitudinal associations between body mass index and symptoms with strong evidence for an association with excess body weight after excluding individuals with the respective depressive symptom at baseline (random-effects meta-analysis) 22](#_Toc107663108)

[Figure S10. Age- and sex-adjusted cross-sectional associations between body mass index and symptoms with strong evidence for an association with excess body weight, stratified by BMI ascertainment (random-effects meta-analysis) 23](#_Toc107663110)

[eTable S6. Serially adjusted cross-sectional associations of body mass index with 9 symptoms of depression (UK Biobank) 24](#_Toc107663112)

[eTable S7. Serially adjusted cross-sectional associations of underweight with 9 symptoms of depression (UK Biobank) 25](#_Toc107663114)

[Appendix 2. Study specific estimates for the association between body mass index and individual depressive symptoms 26](#_Toc107663116)

[FIGURE S11a. Forest plot for the cross-sectional association between overweight and individual depression symptoms (adjusted for age & sex) 26](#_Toc107663117)

[FIGURE S11b. Forest plot for the cross-sectional association between obesity class I and individual depression symptoms (adjusted for age & sex) 30](#_Toc107663118)

[FIGURE S11c. Forest plot for the cross-sectional association between obesity class II and individual depression symptoms (adjusted for age & sex) 34](#_Toc107663119)

[FIGURE S12a. Forest plot for the longitudinal association between overweight and individual depression symptoms at follow-up (adjusted for age, sex, and symptom at baseline) 38](#_Toc107663123)

[FIGURE S12b. Forest plot for the longitudinal association between obesity class I and individual depression symptoms at follow-up (adjusted for age, sex, and symptom at baseline) 39](#_Toc107663125)

[FIGURE S12c. Forest plot for the longitudinal association between obesity class II and individual depression symptoms at follow-up (adjusted for age, sex, and symptom at baseline) 40](#_Toc107663127)

[Appendix 3. Statistical Code 41](#_Toc107663129)

[Statistical Code R: Random-effects meta-analysis of the cross-sectional associations between body mass index categories and individual symptoms of depression (RStudio) 41](#_Toc107663130)

[Statistical Code STATA: Loop for multivariate logistic regression analyses (example) 42](#_Toc107663132)

[References 43](#_Toc107663133)

Appendix 1: Description of cohort studies and supplementary analyses with pooled data

**Costa Rican Longevity and Healthy Aging Study (CRELES)^1^**

The Costa Rican Longevity and Healthy Aging Study (CRELES) is a nationally representative, longitudinal survey which was established in 2005 to explore the health and lifecourse experiences of 2,827 Costa Rican adults aged ≥60 years. Baseline household interviews were conducted between 2004 and 2006, with two-year follow-up interviews in 2007 and 2009. CRELES has collected data on variety of biological, psychological, and sociological measures to assess the causes and consequences of health-related outcomes. Data on height and weight were collected at baseline (2005). Depressive symptoms were measured at baseline and two years later, using the Geriatric Depression Scale.^2^ Written informed consent was obtained prior to the study. Ethical approval was granted by the Ethical Science Committee of the University of Costa Rica (<http://ccp.ucr.ac.cr/creles>).

**English Longitudinal Study of Ageing (ELSA)^3^**

The English Longitudinal Study of Ageing (ELSA) is an ongoing, nationally representative, longitudinal, population study of adults aged ≥50 years living in England. ELSA was established in 2002. Since then, participants have been followed up biannually. Thus far, there have been 9 waves of data collection in ELSA. For the present study, we used data on height and weight from wave 6 (2012/13, baseline). Depressive symptoms were ascertained at baseline and wave 8 (2016/17), using the 8-item version of the Centre for Epidemiological Studies Depression Scale.^4^ Written informed consent was obtained at each data collection wave. Ethical approval was obtained from the London Multi-Centre Research Ethics Committee (<https://www.elsa-project.ac.uk>).

**Midlife in the United States (MIDUS)^5^**

The Midlife in the United States (MIDUS) study is a nationally representative, longitudinal survey of health and well-being of non-institutionalized, English-speaking adults aged 25 - 74 years in the United States. Participant selection was based on random digit dialing conducted between 1995 and 1996. The total original sample (n=7108) includes the main respondents (n=3487), their siblings (n=950), an oversample from five metropolitan areas in the United States (n=757), and a twin subsample (n=1914). The collection of psychosocial data was primarily based on telephone interviews and questionnaires distributed via email to study participants. A follow-up study of the cohort was conducted between 2004-2009 (MIDUS II), which included the *Biomarker Project*. Biological and neurological data of 1,255 respondents was collected from two distinct subsamples: the longitudinal survey sample of 1,054 participants, and the Milwaukee sample of 201 participants who participated in the baseline MIDUS Milwaukee study initiated in 2005. In the present multi-cohort study, we included individuals with complete data on height and weight, covariates, and depressive symptoms. Depressive symptoms were assessed using the 20-item CES-D^4^ questionnaire (<http://www.midus.wisc.edu>).

**Whitehall II^6^**

The Whitehall II study is a prospective cohort study comprising 10,308 men and woman aged 35 to 55 years, who were recruited from 20 London-based civil service departments in 1985. To date, there have been 11 phases of data collection. Since 1985, data has been collected every two to five years, using a wide range of self-report measures and clinical assessments to explore social, psychological, biological and behavioral factors determinants of health, illness, and social inequalities. In the present analyses, we used measures of height and weight at wave 7 (2002/04, baseline). Depressive symptoms were assessed at baseline and three years later (2007/2009) using the 20-item version of the CES-D.^4^ Written informed consent from participants was obtained at each wave of data collection. Ethical approval has been granted by the University College London Medical School Committee on the Ethics of Human Research (<https://www.ucl.ac.uk/iehc/research/epidemiology-public-health/research>).

**Understanding Society (UKHLS)^7^**

Understanding Society, the UK Household Longitudinal Study (UKHLS), is a large-scale prospective cohort study that has followed people from over 40,000 households throughout the UK. It captures a representative sample of people of all age ages and ethnicities. UKHLS was launched in 2009, building on and including data from the British Household Panel Survey (BHPS). To date, there have been 8 waves of data collection. UKHLS has collected a variety of biopsychosocial measures, including information about participants’ social and economic circumstances, psychological well-being, attitudes and beliefs, physical illness, and genetics. Psychosocial data and general health information were collected via annual face-to-face interviews and a self-completion online survey. Biomedical data (e.g., weight, height, blood samples) were collected during the nurse health assessments at wave 2 (2010-12, baseline). Depressive symptoms were ascertained at wave 2 and 3 (2011-13), using the 12-item General Health Questionnaire (GHQ-12).^8^ Written informed consent was obtained at each wave of data collection. UKHLS has been granted ethical approval by the University of Essex Ethics Committee; approval for the nurse health assessment was granted by the National Research Ethics Service approved the nurse health assessment (<https://www.understandingsociety.ac.uk>).

**National Health and Nutrition Survey (NHANES) – 2005/06, 2007/08, 2009/10, 2015/16, and 2017/18^9^**

The National Health and Nutrition Examination Survey (NHANES) is a series of studies designed to ascertain nationally representative information on health and the nutritional status of children and adults in the United States. NHANES was initially established by the National Centre for Health Statistics (NCHS) in the 1960s, and became continuous in 1999, collecting data from a nationally representative sample of approximately 5000 people each year. For the present analyses, we focused on 5 independent NHANES cohorts with complete data on height and weight, covariates, and depressive symptoms from 2005/06, 2007/08, 2009/10, 2015/16, and 2017/18. Depressive symptoms were measured using 9 items of the Patient Health Questionnaire (PHQ-9).^10^ Written informed consent was provided prior to each study. Ethical approval was granted by the NCHS Research Ethics Review Board (ERB) (<https://wwwn.cdc.gov/Nchs/Nhanes/2009-2010/CRP_F.htm>).

**Social Environment and Biomarkers of Aging Study (SEBAS)^11^**

The Social Environment and Biomarkers of Ageing Study (SEBAS) is a nationally representative, longitudinal survey of both middle-aged and older adults living in Taiwan. The first wave of data collection was conducted in 2000 and participants were followed up in 2006. SEBAS provides a rich variety of social, environmental, psychological and biological data. Baseline body mass index was computed from participants’ height and weight. Depressive symptoms were assessed at baseline and six years later, using a 10-item version of the CES-D.^4^ SEBAS was approved by ethical committees in Taiwan, at Georgetown University, and at Princeton University. Written informed consent was obtained prior to the study. More information about the Social Environment and Biomarkers of Aging Study can be found at the Georgetown University Centre for Populations and Health website (<https://cph.georgetown.edu/research/taiwan/>).

**Health and Retirement Study (HRS)^12^**

The Health and Retirement Study (HRS) is a nationally representative, longitudinal, biannual, population-based cohort study of approximately 20,000 people aged ≥51 years in the United States. HRS was launched in 1992 by the University of Michigan and is funded by the National Institute on Aging. Since then, HRS has collected information about income, work, assets, pension plans, health insurance, disability, physical health and functioning, mental health, cognitive functioning, and health care expenditures (<https://hrs.isr.umich.edu/about>). Height and weight were measured at baseline and body mass index was computed. The 20-item version of the CES-D^4^ to assess depressive symptoms. Participants provided written informed consent prior to the study, and ethical approval was granted by the University of Michigan Institutional Review Board.

**The Irish Longitudinal Study of Ageing (TILDA)^13^**

The Irish Longitudinal Study of Ageing (TILDA) is a prospective cohort study exploring the economic, social, age-, and health-related experiences of community-dwelling adults aged ≥50 years in the Republic of Ireland. The first wave of data collection was conducted between October 2009 and July 2011 in a representative sample of 8,175 adults. Sampling was based on a three-stage selection process using the Geodirectory (i.e., an extensive list of all addresses registered in Ireland) as a sampling frame. First, subdivisions of electoral districts stratified by socio-economic status, age, and geographical location were used as the primary sampling unit. Second, a random probability sample of 40 addresses was selected from within each cluster, resulting in an initial sample of 25,600 addresses. The third stage involved asking all adults aged ≥50 years of selected households to participate. Participants completed a computer assisted personal interview (CAPI) and a self-administered questionnaire and were then invited to attend one of two test centers for the collection of physical samples and bio-medical measures. Height and weight were used to compute body mass index. Depressive symptoms were assessed using the 20-item version of the CES-D.^4^ All participants provided written informed consent prior to the study. Ethical approval was granted by the Research Ethics Committee of Trinity College Dublin (<https://tilda.tcd.ie>).

**The National Social Life, Health, and Aging Project (NSHAP)^14^**

The National Social Life, Health, and Aging Project (NSHAP) is a nationally representative, longitudinal cohort study of community-dwelling older adults in the United States. NSHAP aims to examine the interactions among and relationships between physical health and illness, mental health, medication use, health behaviours, cognitive functioning, social relationships, and sexuality. Thus far, there have been three waves of data collection. The first wave of NSHAP was conducted between 2005 and 2006 by NORC and Principal Investigators at the University of Chicago, completing more than 3,000 interviews with a sample of adults born between 1920 and 1947 (aged 57 to 85 at the time of Wave 1 interview). Approximately 3,400 interviews were conducted for wave 1 with wave 2 respondents, wave 1 non-interviewed respondents, and their spouses or cohabiting romantic partners between 2010 and 2011. Wave 3 was conducted from September 2015 to November 2016, including 2,409 wave 2 respondents, and a refresher cohort consisting of adults born between 1948 and 1965 together with their spouses or romantic partners. A total 4,777 respondents were interviewed in Wave 3. Data on body mass index was derived from participants’ height and weight. Depressive symptoms were ascertained from the 11 items of the CES-D.^4^ All participants gave their written informed consent prior to participation in the study. The study protocol was approved by NORC, the Institutional Review Boards of the University of Chicago, and the University of Arizona.

**The Mexican Health and Aging Study (MHAS)^15^**

The Mexican Health and Aging Study (MHAS) is a nationally representative, longitudinal cohort study of adults aged ≥50 years living in both urban and rural areas in Mexico. MHAS was designed to explore health and ageing-related biopsychosocial mechanisms and outcomes. To date, five waves of data collection have been conducted; a baseline wave of adults born in 1951 or earlier was conducted in 2001 with follow-up interviews held in 2003, 2012, 2015, and 2018. Data on height and weight were collected to calculate participants’ body mass index. Depressive symptoms were ascertained from 9-items of the CES-D.^4^ All participants provided written informed consent prior to each wave of data collection. Ethical approval for MHAS study protocols and instruments were granted by the Institutional Review Board or Ethics Committee of the University of Texas Medical Branch, the Instituto Nacional de Estadistica y Geografia (INEGI) in Mexico, and the Instituto Nacional de Salud Publica (INSP) in Mexico (<http://www.mhasweb.org>).

Figure S1. Selection of studies for individual-participant pooled analysis of the association between excess body weight and individual symptoms of depression & selection of participants in the UK Biobank


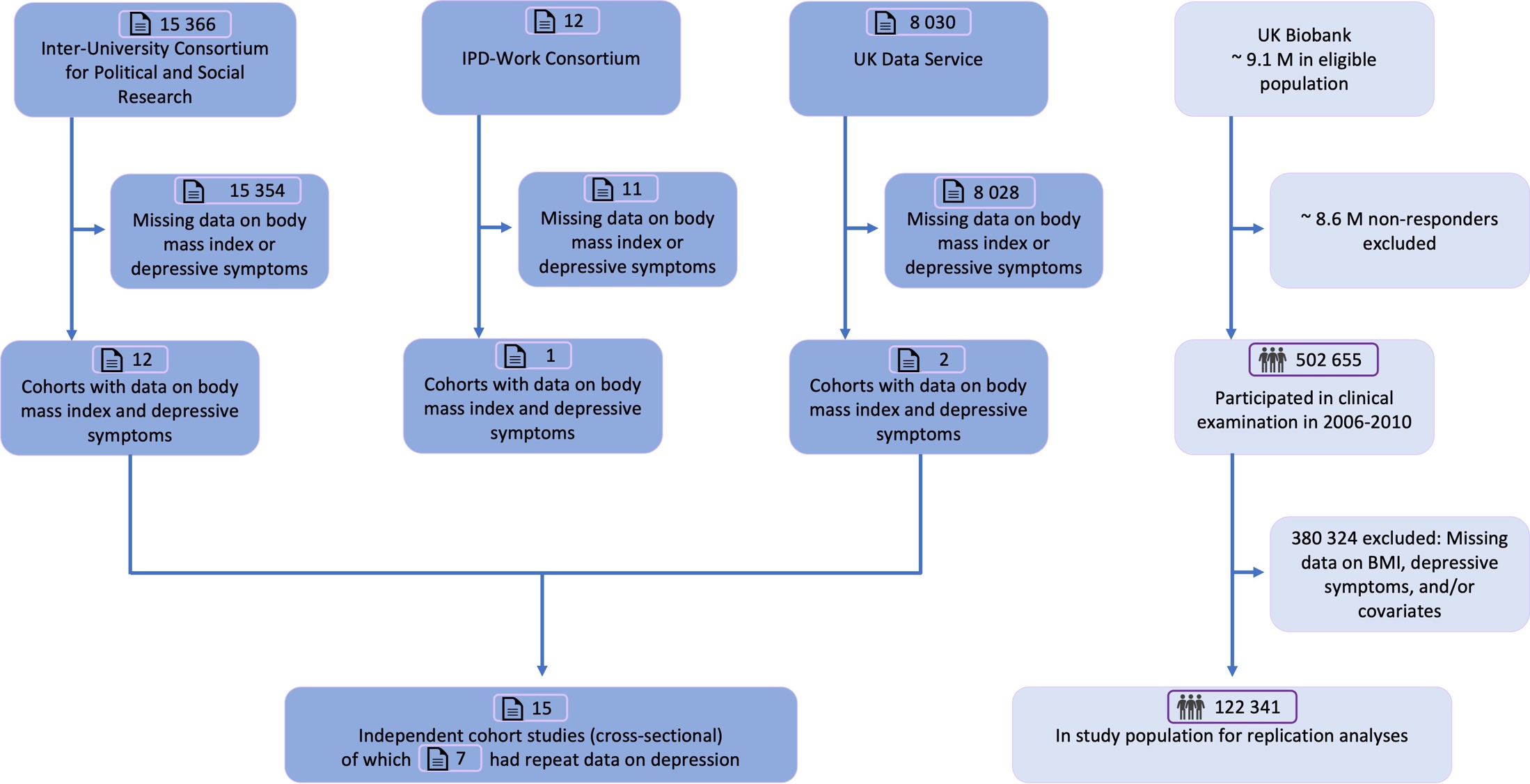


eTable S1. Baseline characteristics of the included cohorts


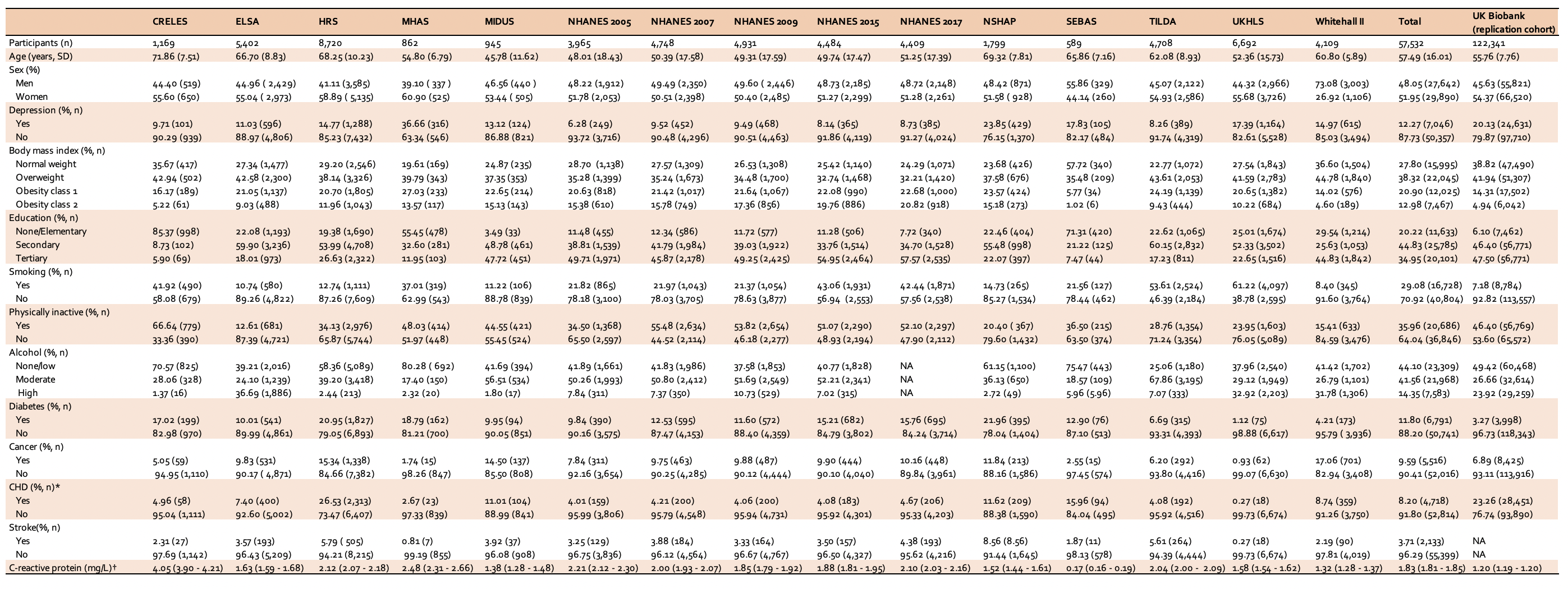


* In UK Biobank, this refers to the presence or absence of any heart disease, including heart attack, angina, stroke, and high blood pressure.

† Geometric mean CRP (mg/L) [mean (CI)]

eTable S2. Study characteristics and method of outcome (depressive symptoms) ascertainment

| **Study** | **Baseline (year)** | **Country** | **Number of participants** | **Percentage (number) of women** | **Mean age at baseline (years)** | **Method of body mass index ascertainment** | **Method of depression symptoms ascertainment** | **Cut-off depression measures** | **Question & Response scale** | **Harmonisation cut-off (items)** |
| --- | --- | --- | --- | --- | --- | --- | --- | --- | --- | --- |
| SEBAS | 2000 | Taiwan | 589 | 44.14 (260) | 65.86 (7.16) | Measured | Centre for Epidemiological Studies Depression Scale | ≥ 10 | How often during the past week did you feel this way? Rarely/none of the time = 0; some or a little of the time = 1; occasionally or moderate amount of time = 2; most or all of the time = 3 | >2 |
| MHAS * | 2012 | Mexico | 862 | 60.90 (525) | 54.80 (6.79) | Self-reported | Centre for Epidemiological Studies Depression Scale | ≥ 4 | Please tell me if each of the following was true for you much of the time during the past week.  Yes = 1; No = 0 | =1 |
| MIDUS | 2004-2009 | USA | 945 | 86.88 (821) | 45.78 (11.62) | Measured | Centre for Epidemiological Studies Depression Scale | ≥ 16 | How often during the past week did you feel this way? Rarely/none of the time = 0; some or a little of the time = 1; occasionally or moderate amount of time = 2; most or all of the time = 3 | >2 |
| CRELES | 2005 | Costa Rica | 1169 | 55.60 (650) | 71.86 (7.51) | Measured | Geriatric Depression Scale | ≥ 10 | Please tell me if each of the following was true for you much of the time during the past week.  Yes = 1; No = 0 | =1 |
| NSHAP * | 2005-2006 | USA | 1799 | 51.58 (928) | 69.32 (7.81) | Measured | Centre for Epidemiological Studies Depression Scale | ≥ 9 | How often during the past week did you feel this way? Rarely/none of the time = 0; some or a little of the time = 1; occasionally or moderate amount of time = 2; most or all of the time = 3 | >2 |
| NHANES 2005 | 2005-2006 | USA | 3965 | 51.78 (2053) | 48.01 (18.43) | Measured | Depression Screener Questionnaire | ≥ 10 | Over the last 2 weeks, how often have you been bothered by the following problems: […] Not at all = 0; several days =1; more than half of the days =2; nearly every day = 3 | >2 |
| Whitehall II * | 2002-2004 | UK | 4109 | 26.92 (1106) | 60.80 (5.89) | Measured | Centre for Epidemiological Studies Depression Scale | ≥ 16 | How often during the past week did you feel this way? Rarely/none of the time = 0; some or a little of the time = 1; occasionally or moderate amount of time = 2; most or all of the time = 3 | >2 |
| NHANES 2017 | 2017-2018 | USA | 4409 | 51.28 (2261) | 51.25 (17.39) | Measured | Depression Screener Questionnaire | ≥ 10 | Over the last 2 weeks, how often have you been bothered by the following problems: […] Not at all = 0; several days =1; more than half of the days =2; nearly every day = 3 | >2 |
| NHANES 2015 | 2015-2016 | USA | 4484 | 51.27 (2299) | 49.74 (17.47) | Measured | Depression Screener Questionnaire | ≥ 10 | Over the last 2 weeks, how often have you been bothered by the following problems: […] Not at all = 0; several days =1; more than half of the days =2; nearly every day = 3 | >2 |
| TILDA * | 2009-2011 | Ireland | 4708 | 54.93 (2586) | 62.08 (8.93) | Self-reported | Centre for Epidemiological Studies Depression Scale | ≥ 16 | How often during the past week did you feel this way? Rarely/none of the time = 0; some or a little of the time = 1; occasionally or moderate amount of time = 2; most or all of the time = 3 | >2 |
| NHANES 2007 | 2007-2008 | USA | 4748 | 50.51 (2398) | 50.39 (17.58) | Measured | Depression Screener Questionnaire | ≥ 10 | Over the last 2 weeks, how often have you been bothered by the following problems: […] Not at all = 0; several days =1; more than half of the days =2; nearly every day = 3 | >2 |
| NHANES 2009 | 2009-2010 | USA | 4931 | 50.40 (2485) | 49.31 (17.59) | Measured | Depression Screener Questionnaire | ≥ 10 | Over the last 2 weeks, how often have you been bothered by the following problems: […] Not at all = 0; several days =1; more than half of the days =2; nearly every day = 3 | >2 |
| ELSA * | 2012-2013 | UK | 5402 | 55.04 (2973) | 66.70 (8.83) | Measured | Centre for Epidemiological Studies Depression Scale | ≥ 4 | Please tell me if each of the following was true for you much of the time during the past week.  Yes = 1; No = 0 | =1 |
| UKHLS * | 2010-2012 | UK | 6692 | 55.68 (3726) | 52.36 (15.73) | Self-reported | General Health Questionnaire | ≥ 4 | Have you recently been feeling […] Not at all = 0; no more than usual = 1; rather more than usual =2; much more than usual = 3 | >2 |
| HRS * | 2006-2008 | USA | 8720 | 58.89 (5135) | 68.25 (10.23) | Self-reported | Centre for Epidemiological Studies Depression Scale | ≥ 4 | Please tell me if each of the following was true for you much of the time during the past week.  Yes = 1; No = 0 | =1 |
| **Total** |  |  | **57532** | **51.95 (29890)** | **57.49 (16.01)** |  |  |  |  |  |
| **UK Biobank** | 2006 | UK | 122341 | 54.4 (66520) | 55·8 (7.8). | Self-reported | Patient Health Questionnaire (PHQ-9) |  | Over the last 2 weeks, how often have you been bothered by the following problems: […] Not at all = 0; several days =1; more than half of the days =2; nearly every day = 3 | >2 |

eTable S3. Robust, serially adjusted cross-sectional associations of body mass index with 6 top symptoms of depression (random-effects meta-analysis of cohort studies)


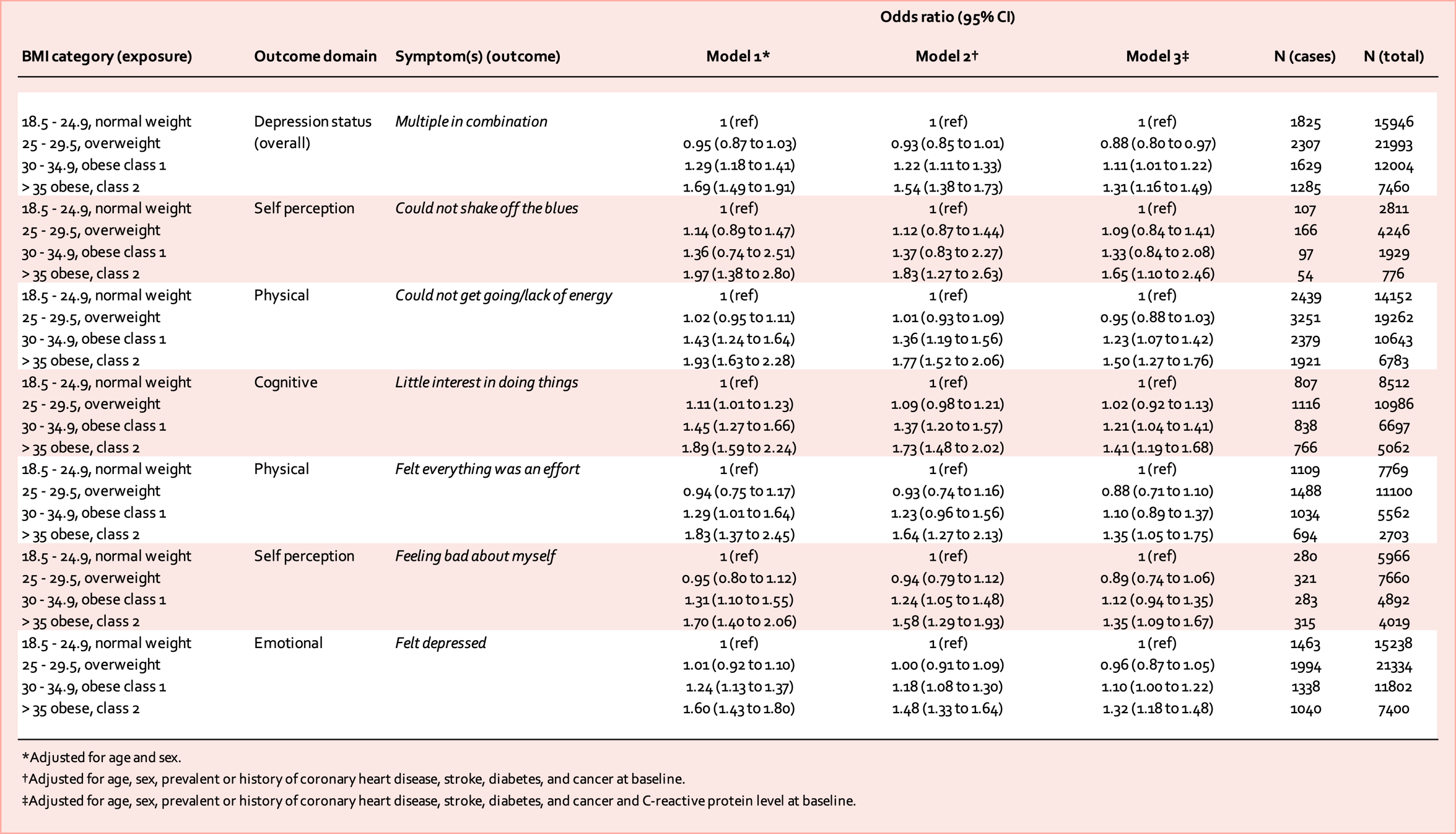


Figure S2a. Serially adjusted cross-sectional associations of body mass index with emotional symptoms of depression (random-effects meta-analysis)

**
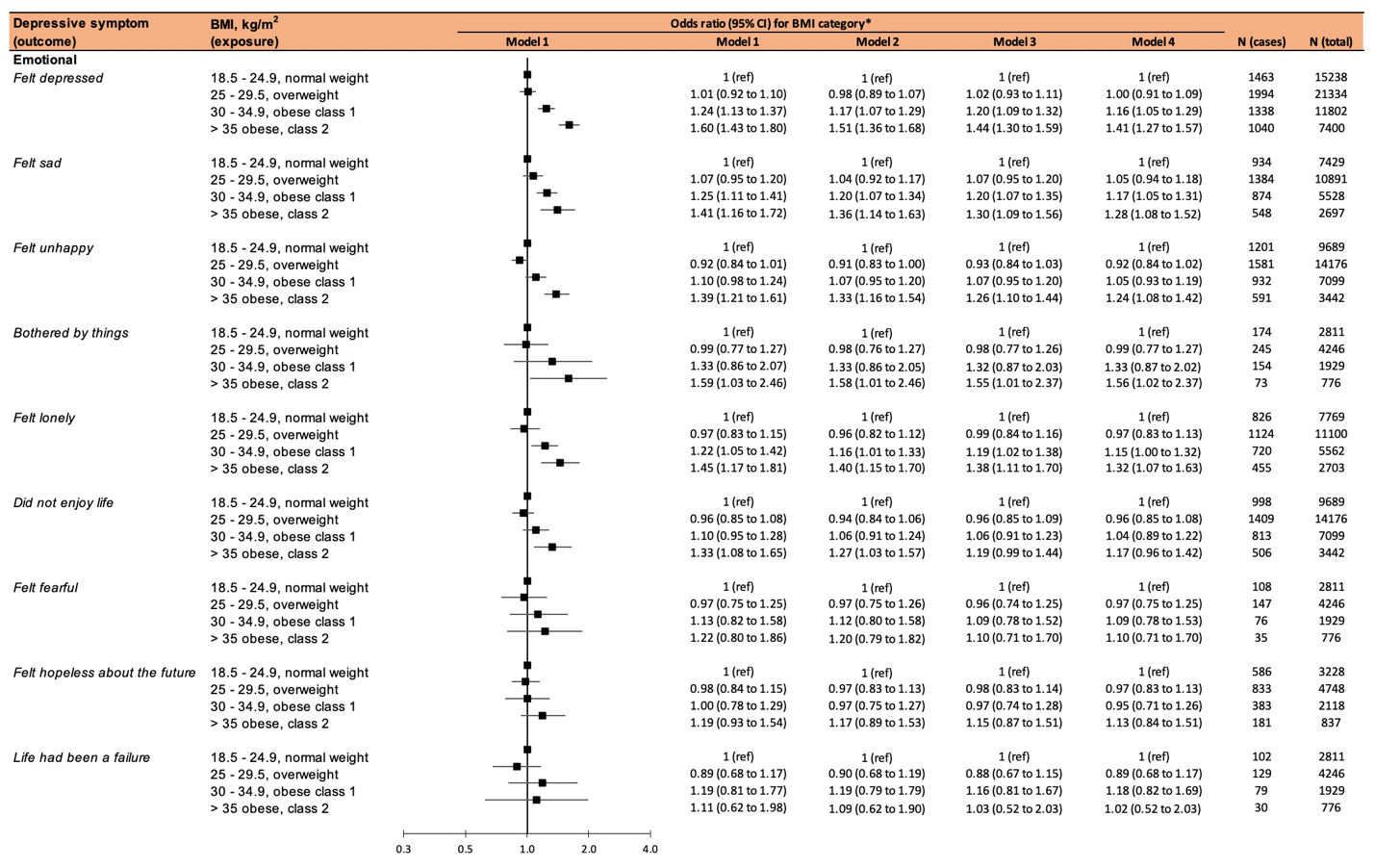
**

*Model 1 is adjusted for age and sex. Model 2 is adjusted for age, sex, and education. Model 3 is adjusted for age, sex, and behavioral factors. Model 4 is adjusted for age, sex, education, and behavioral factors.

Figure S2b. Serially adjusted cross-sectional associations of body mass index with physical symptoms of depression (random-effects meta-analysis)

**
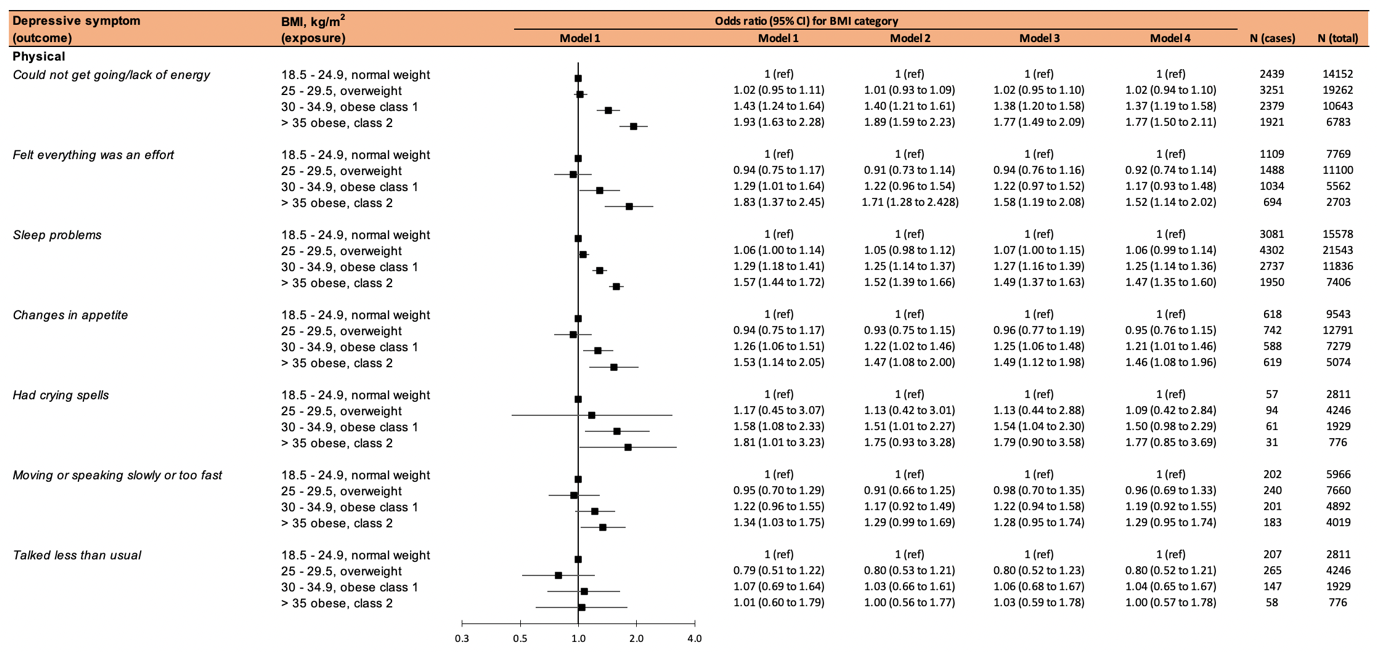
**

*Model 1 is adjusted for age and sex. Model 2 is adjusted for age, sex, and education. Model 3 is adjusted for age, sex, and behavioral factors. Model 4 is adjusted for age, sex, education, and behavioral factors.

Figure S2c. Serially adjusted cross-sectional associations of body mass index with cognitive, self-perception-related, and self-harm-related symptoms of depression (random-effects meta-analysis)

**
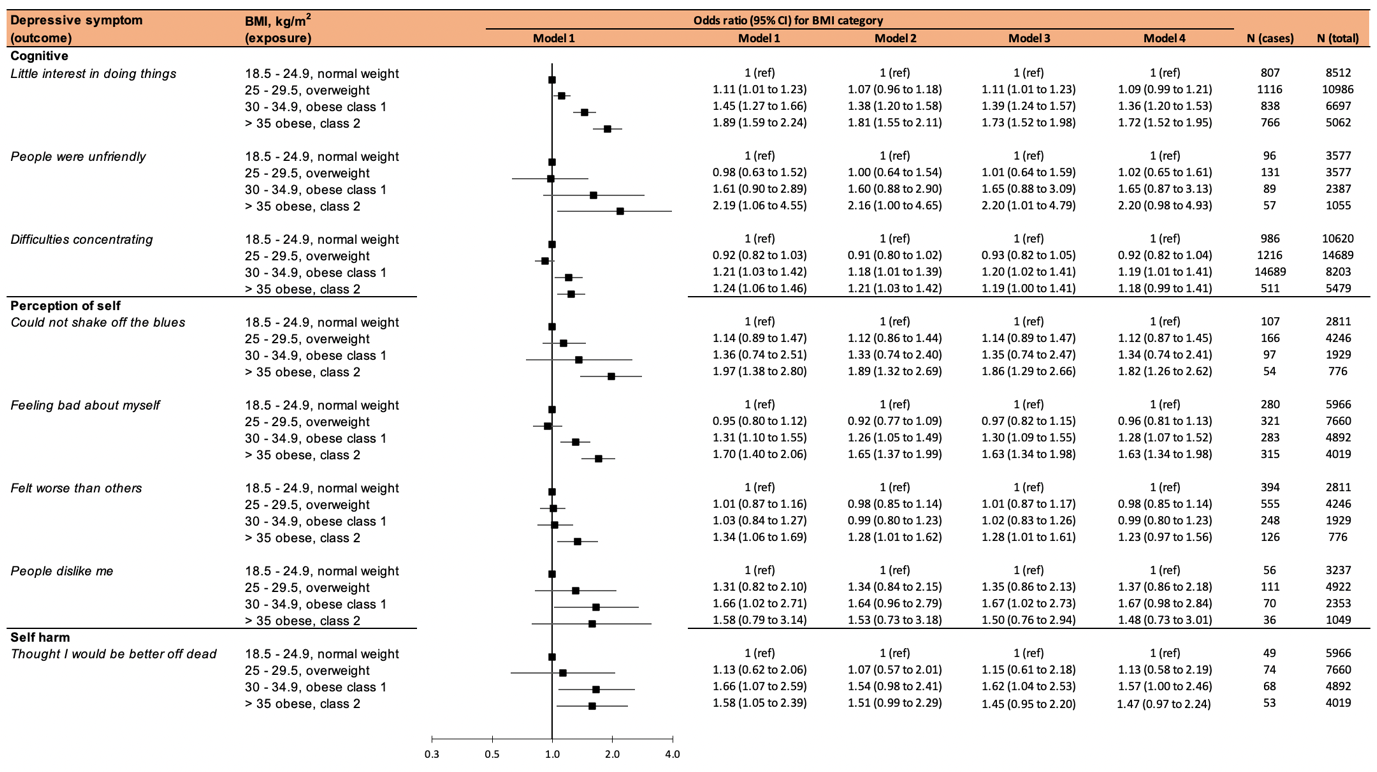
**

*Model 1 is adjusted for age and sex. Model 2 is adjusted for age, sex, and education. Model 3 is adjusted for age, sex, and behavioral factors. Model 4 is adjusted for age, sex, education, and behavioral factors.

eTable S4. Cross-sectional associations of body mass index with individual symptoms of depression adjusted for chronic disease and C-reactive protein (random-effects meta-analysis)

**
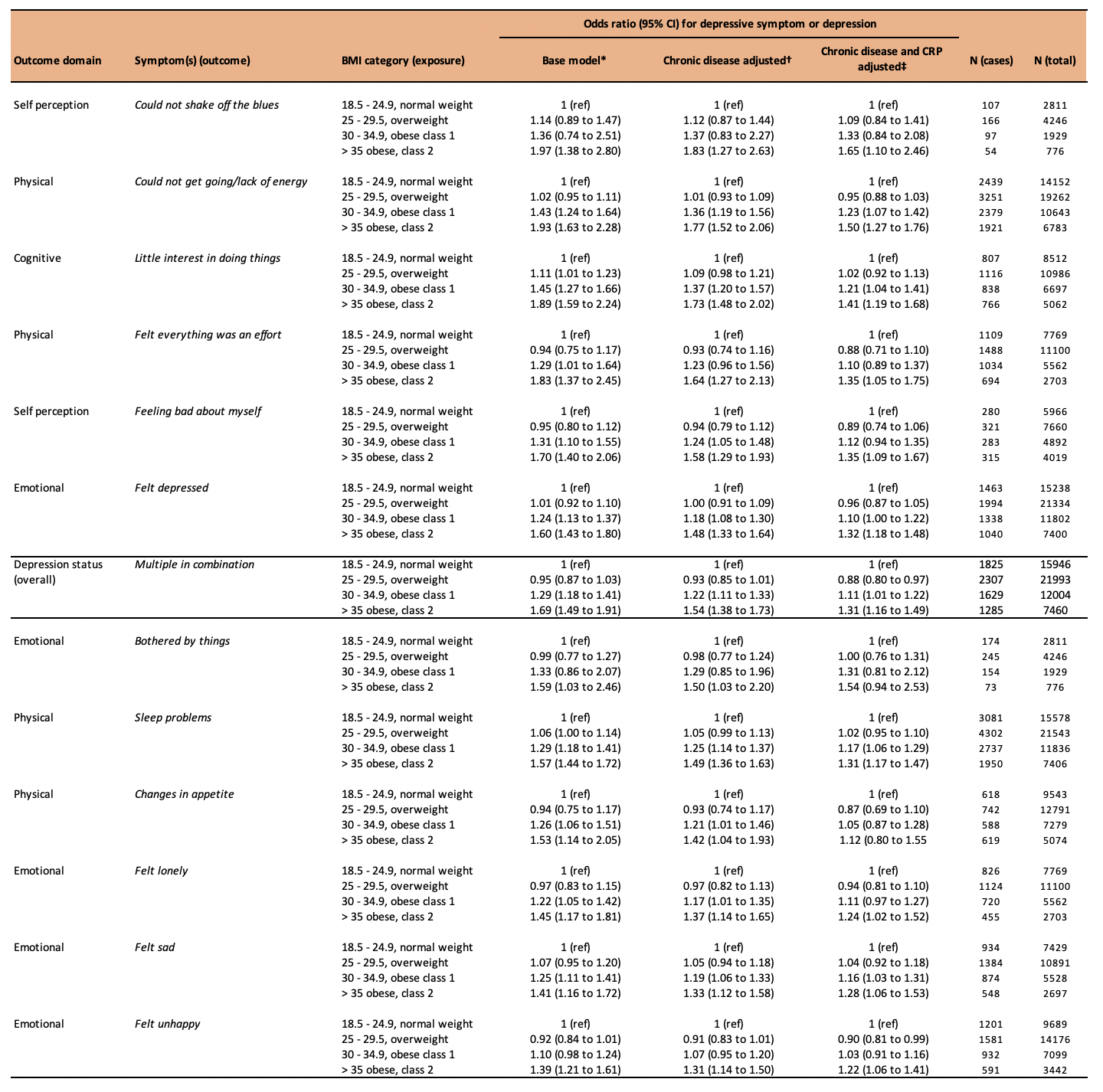
**

*Adjusted for age and sex; †Adjusted for age, sex, chronic illnesses (i.e., coronary heart disease, stroke, cancer, diabetes); ‡Adjusted for age, sex, chronic illnesses, and C-reactive protein.

Note: These symptom-specific associations were robust in cross-sectional analyses adjusting for socio-demographic covariates, education, and behavioral factors.

**Figure S3. Age-adjusted sex-stratified and sex-adjusted age-stratified cross-sectional associations of body mass index with 4 individual symptoms (random-effects meta-analysis of cohort studies)**
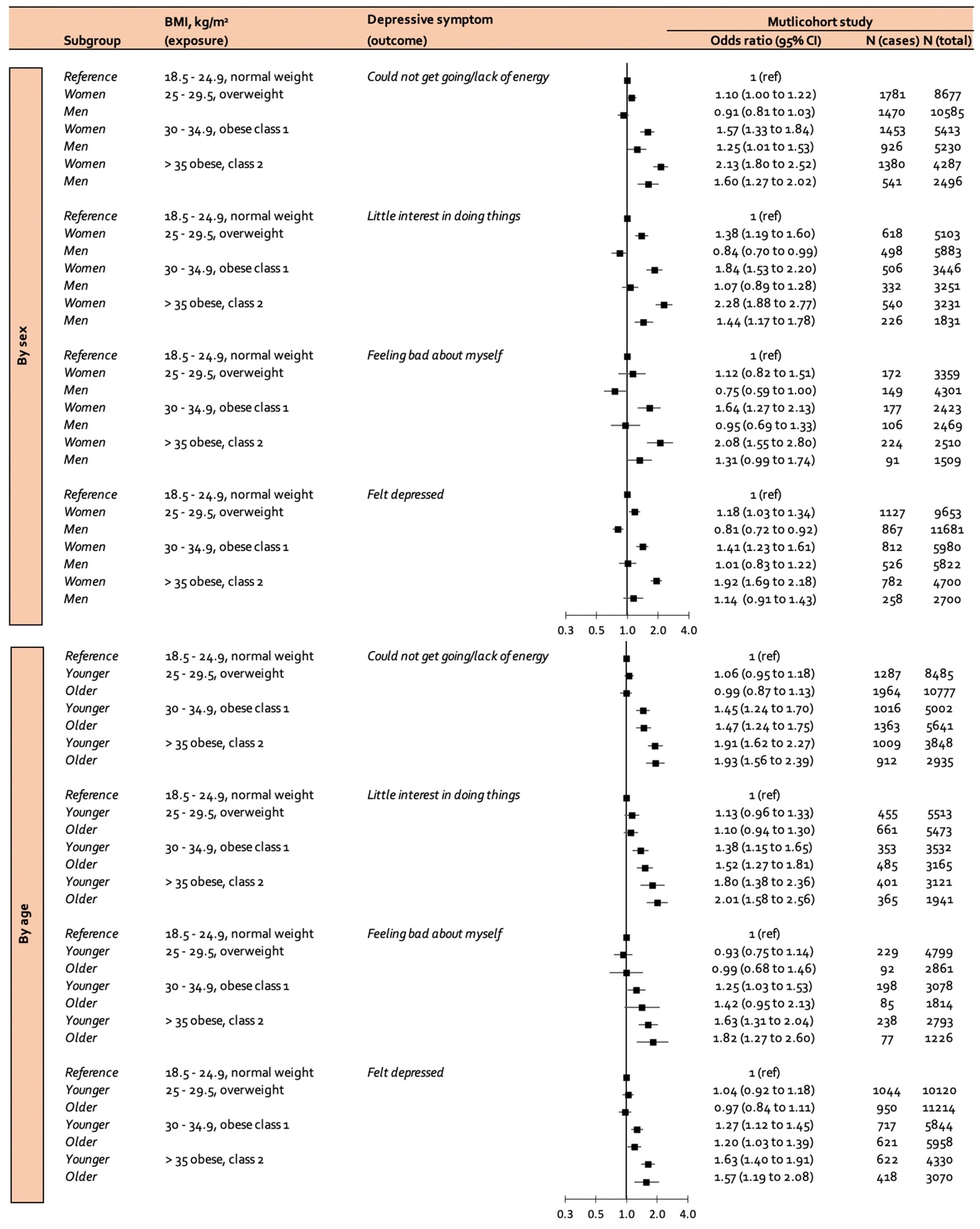


Figure S4. Age- and sex-adjusted cross-sectional associations of body mass index with 6 top symptoms among depressed individuals (random-effects meta-analysis)


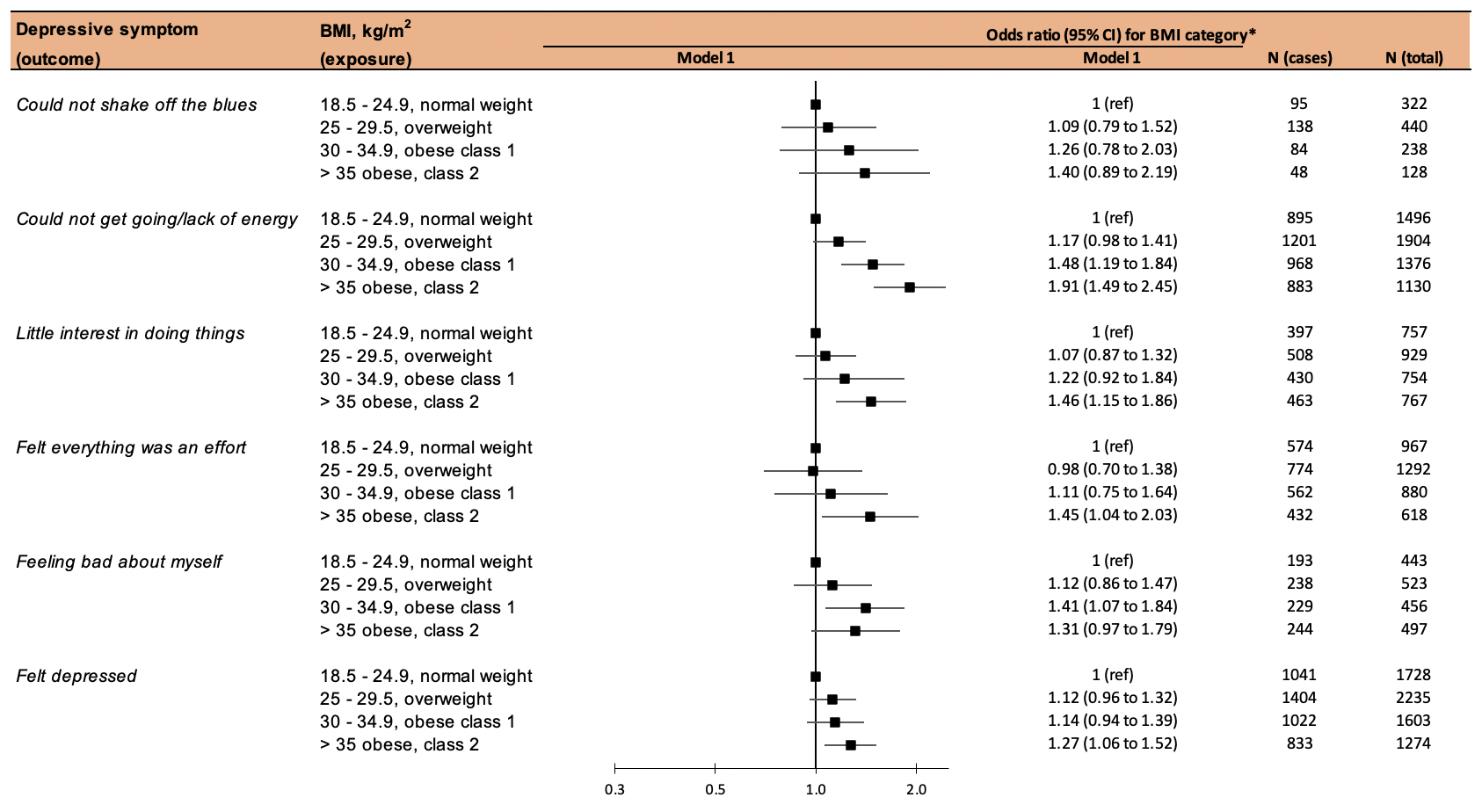


*Adjusted for age & sex.

Note: These symptom-specific associations were robust to adjustment for socio-demographic, behavioral, and chronic illness-related factors, including C-reactive protein.

Figure S5. Age- and sex-adjusted longitudinal associations of body mass index with 5 top symptoms and overall depression status after excluding participants with depression at baseline (random-effects meta-analysis)


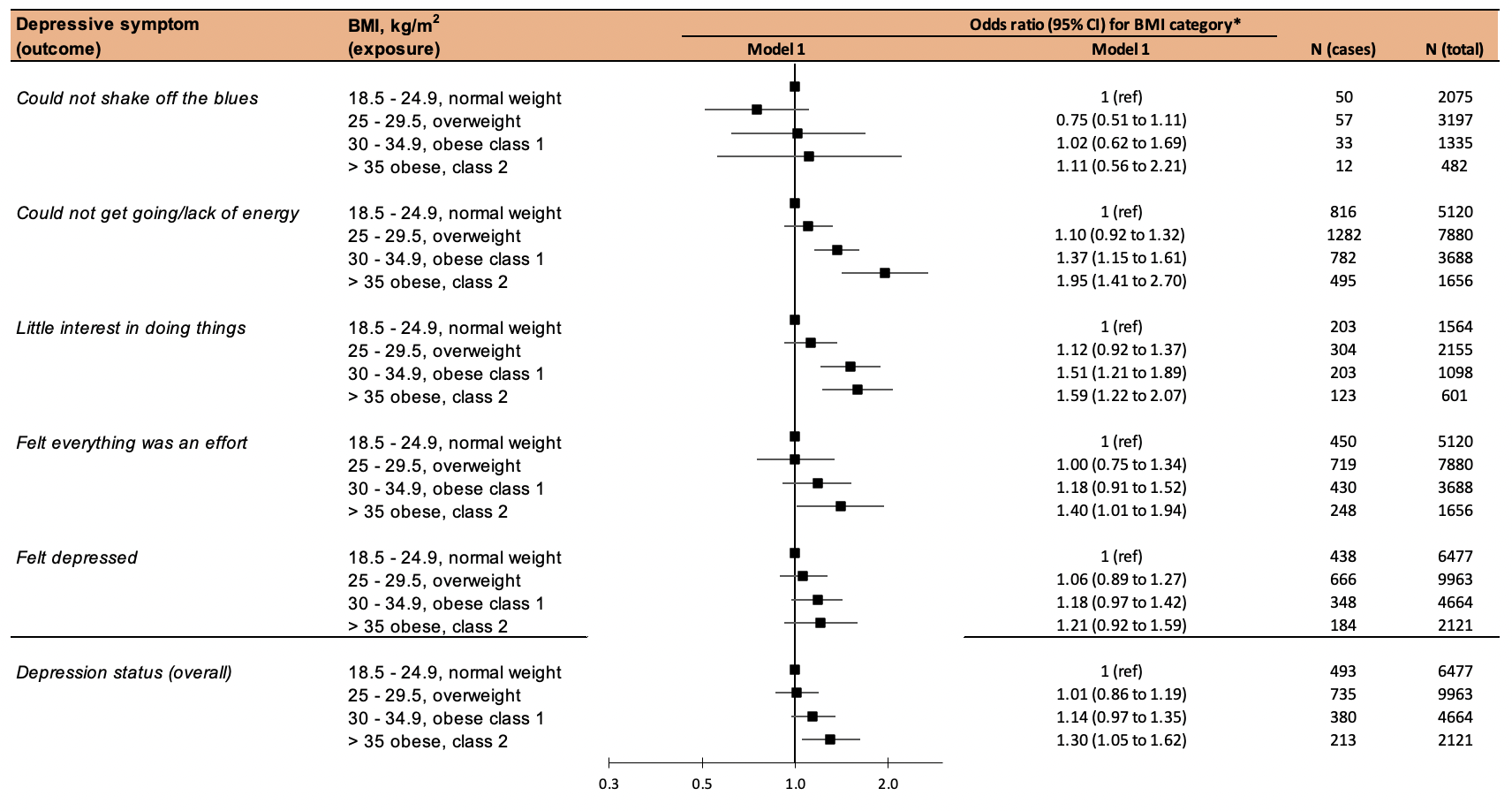


Note: Individuals with depression at baseline were excluded to control for the potential influence of anti-depressant medication. No longitudinal data were available for the symptom "feeling bad about myself". For the symptom “little interest in doing things”, data was available from one cohort only (HRS).

*Model 1: odds ratios adjusted for age, sex, and baseline depression symptom.

eTable S5. Serially adjusted cross-sectional associations of body mass index with 4 symptoms with strong evidence against an association with excess body weight (random-effects meta-analysis)

**
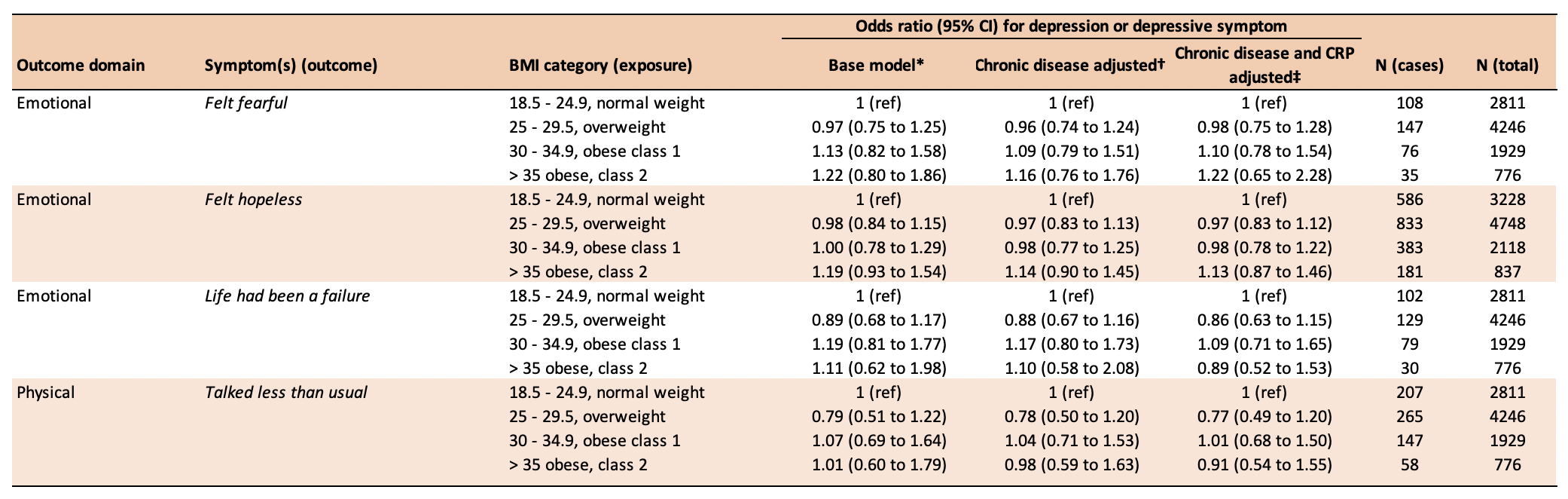
**

*Adjusted for age and sex; †Adjusted for age, sex, chronic illnesses (i.e., coronary heart disease, stroke, cancer, diabetes); ‡Adjusted for age, sex, chronic illnesses, and C-reactive protein

Figure S6. Age-adjusted sex-stratified cross-sectional associations of body mass index with 4 symptoms with strong evidence against an association with excess body weight (random-effects meta-analysis)

**
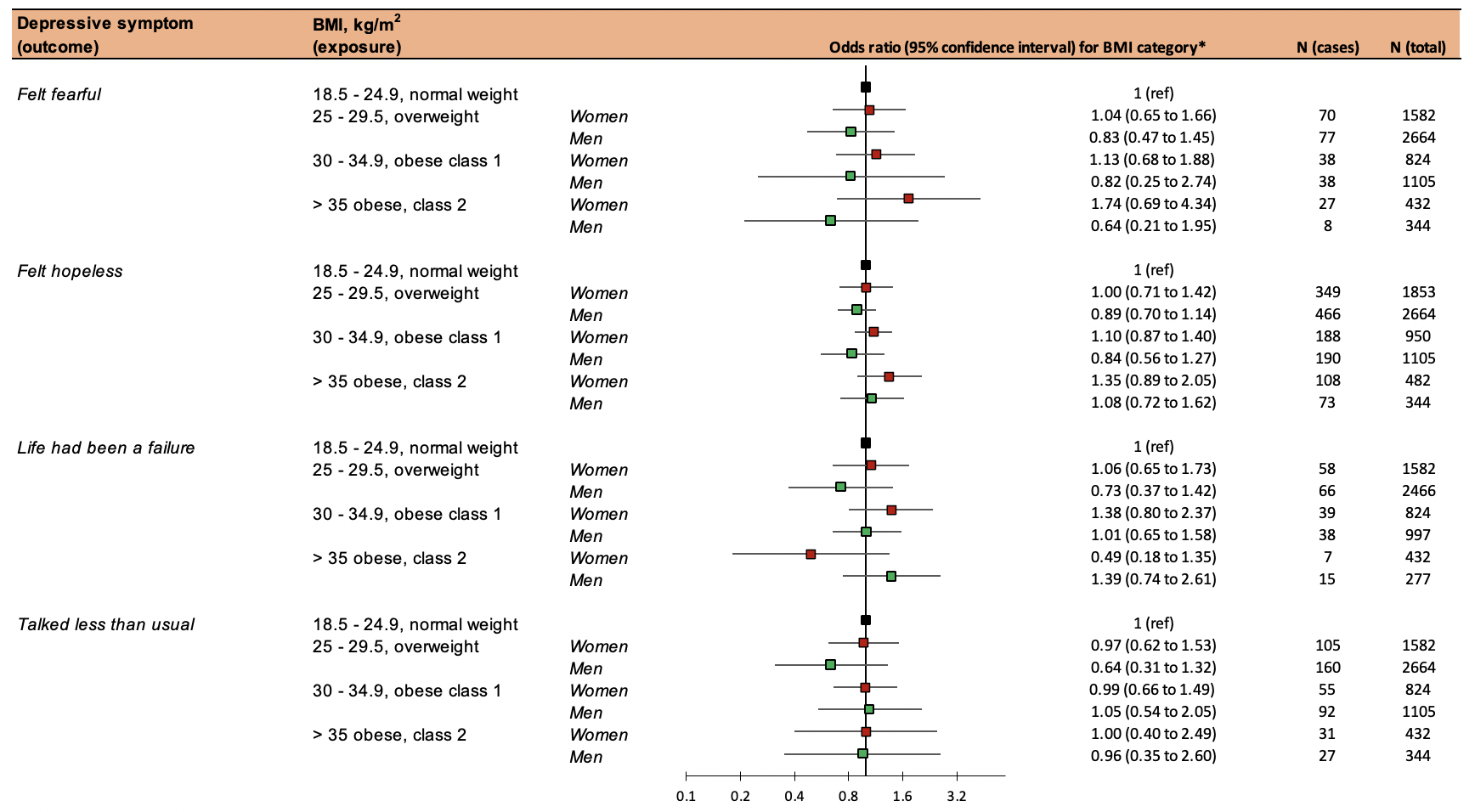
**

* Odds ratios adjusted for age.

Figure S7. Sex-adjusted age-stratified cross-sectional associations of body mass index with 4 symptoms with strong evidence against an association with excess body weight (random-effects meta-analysis)

**
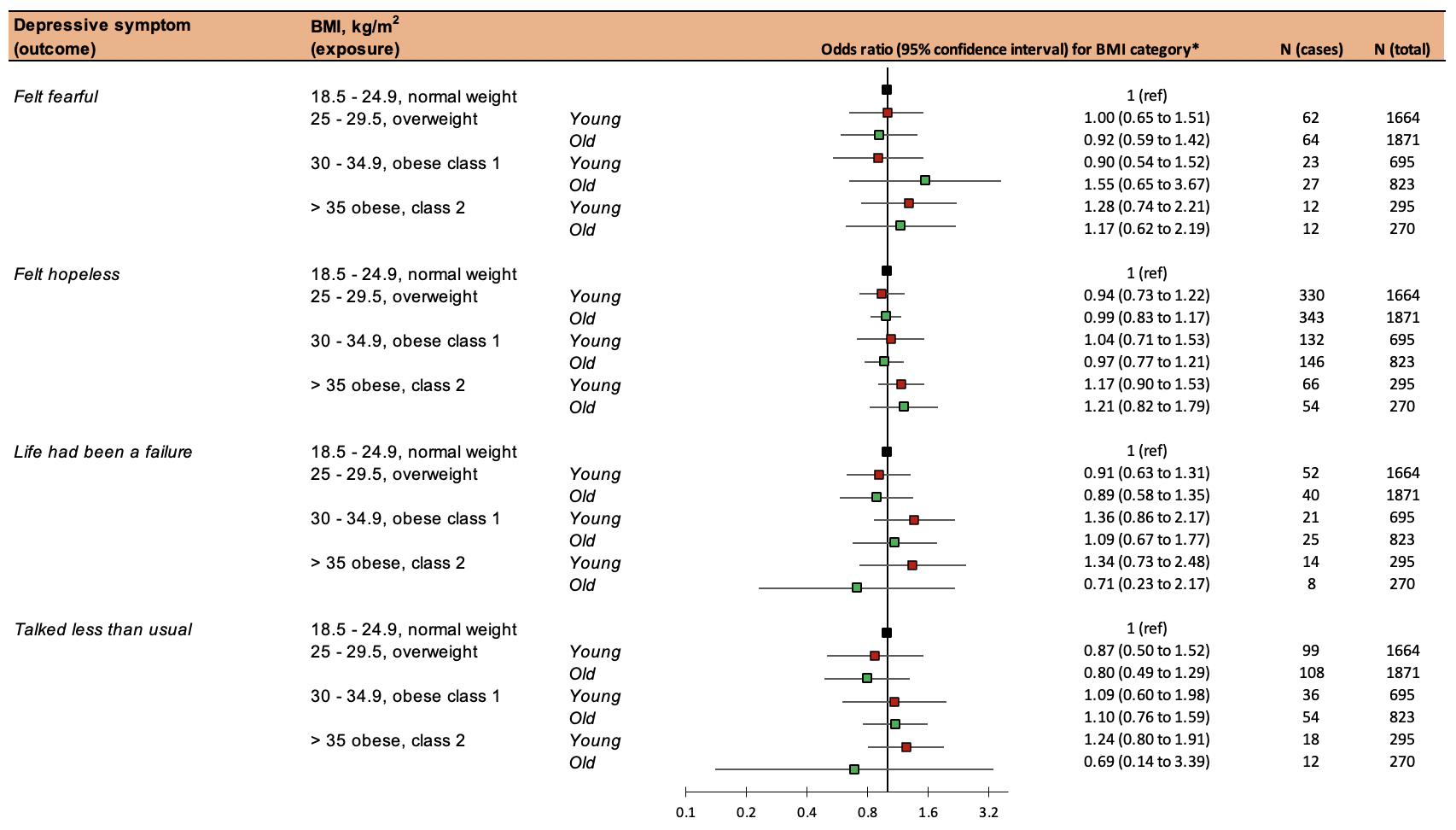
**

* Odds ratios adjusted for sex.

Figure S8. Age- and sex-adjusted longitudinal associations of body mass index with symptoms with strong evidence against an association with excess body weight (random-effects meta-analysis)


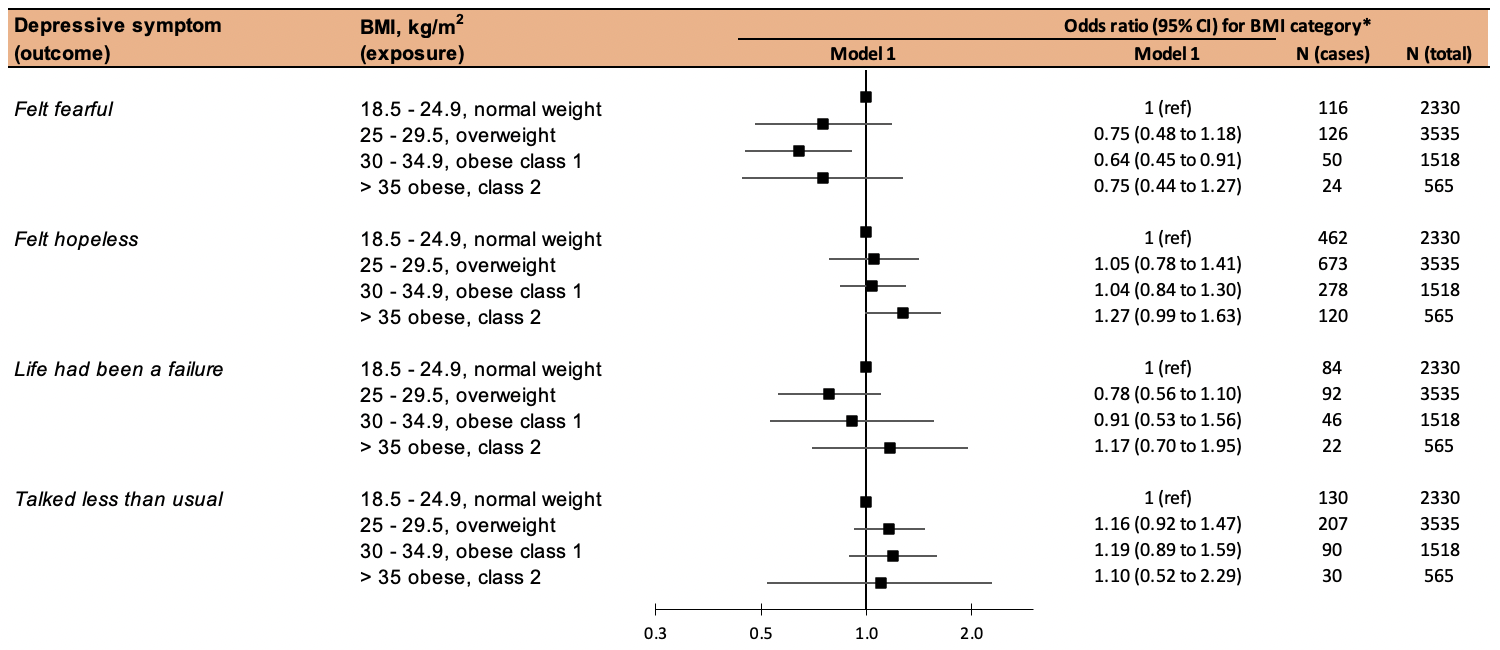


*Model 1: odds ratios adjusted for age, sex, and baseline depression symptom.

Figure S9. Age- and sex-adjusted longitudinal associations between body mass index and symptoms with strong evidence for an association with excess body weight after excluding individuals with the respective depressive symptom at baseline (random-effects meta-analysis)


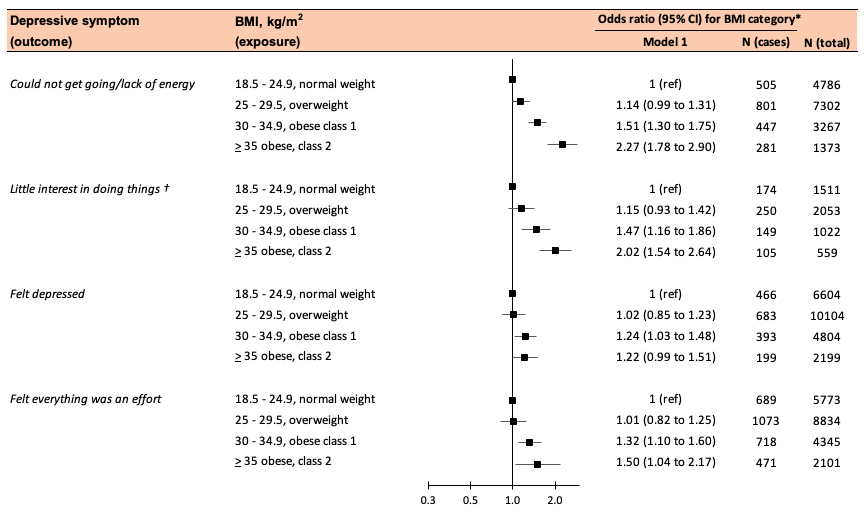


| * Model 1 is adjusted for age, sex. No longitudinal data were available for the symptom "feeling bad about myself". | | | | | | | | | | |
| --- | --- | --- | --- | --- | --- | --- | --- | --- | --- | --- |
| † Data available from one cohort only (HRS). |  |  |  |  |  |  |  |  |  |  |

Figure S10. Age- and sex-adjusted cross-sectional associations between body mass index and symptoms with strong evidence for an association with excess body weight, stratified by BMI ascertainment (random-effects meta-analysis)


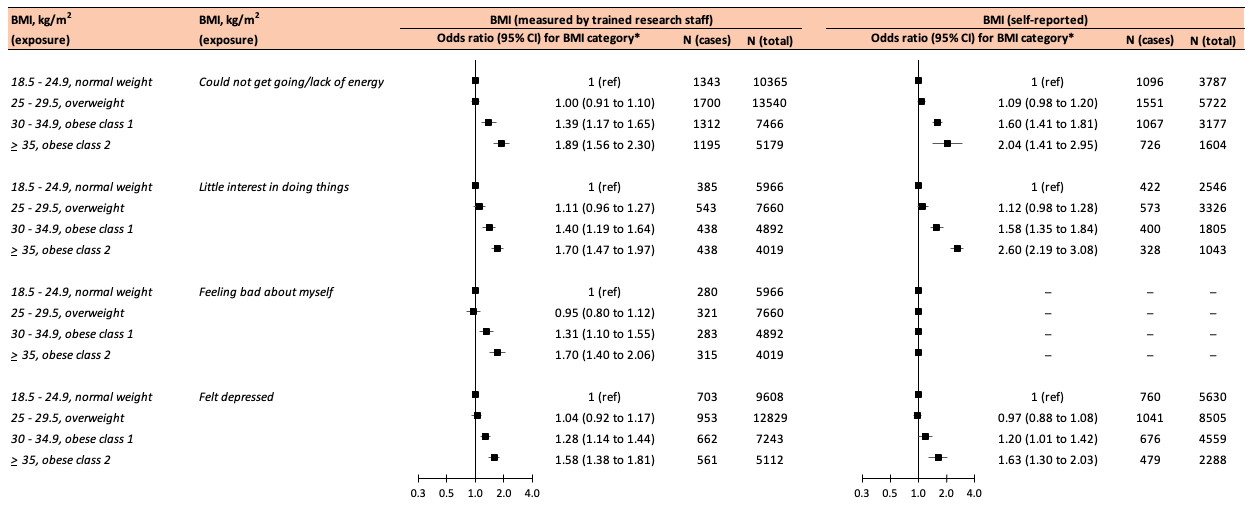


*Odds ratios adjusted for age and sex.

eTable S6. Serially adjusted cross-sectional associations of body mass index with 9 symptoms of depression (UK Biobank)


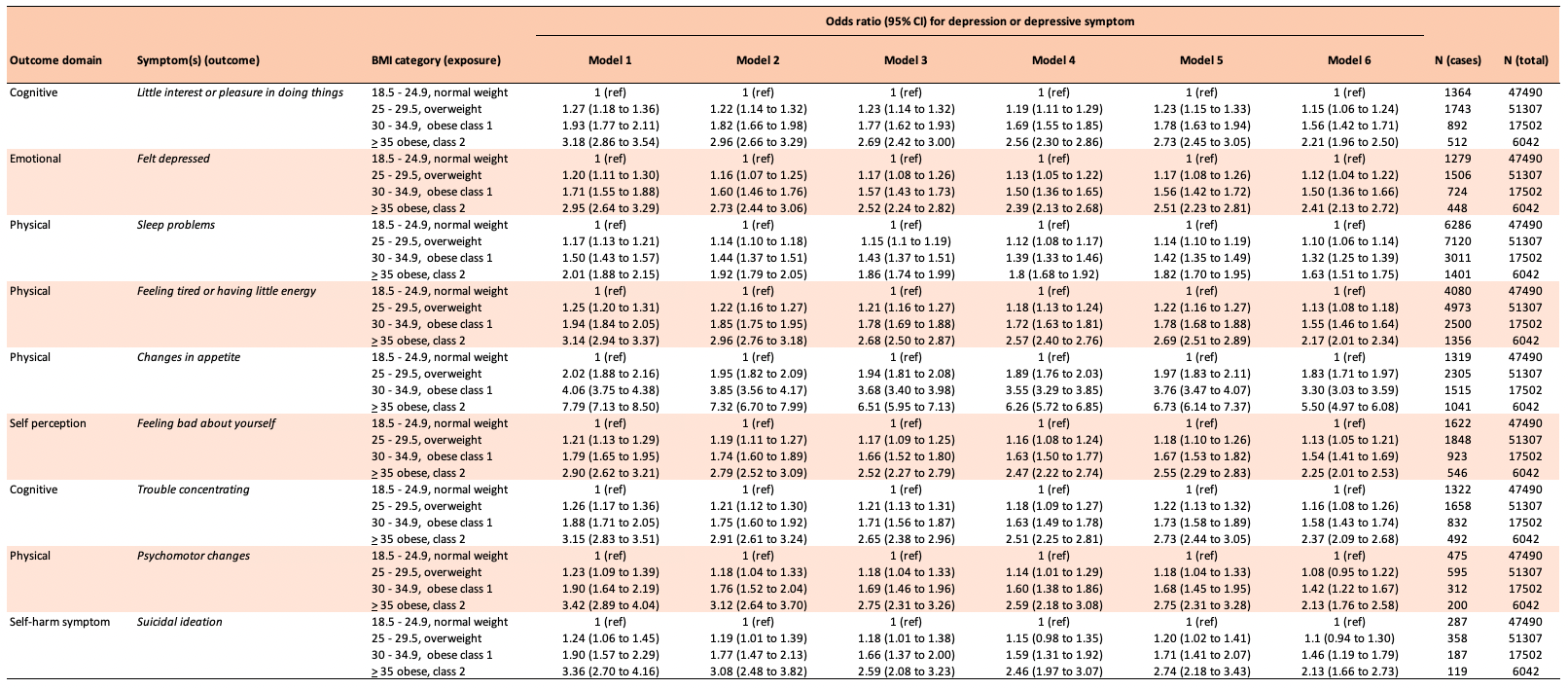


Model 1 is adjusted for age and sex. Model 2 is adjusted for age, sex, and education. Model 3 is adjusted for age, sex, and behavioral factors. Model 4 is adjusted for age, sex, education, and behavioral factors. Model 5 is adjusted for age, sex, and chronic illnesses. Model 6 is adjusted for age, sex, chronic illnesses, and C-reactive protein.

eTable S7. Serially adjusted cross-sectional associations of underweight with 9 symptoms of depression (UK Biobank)


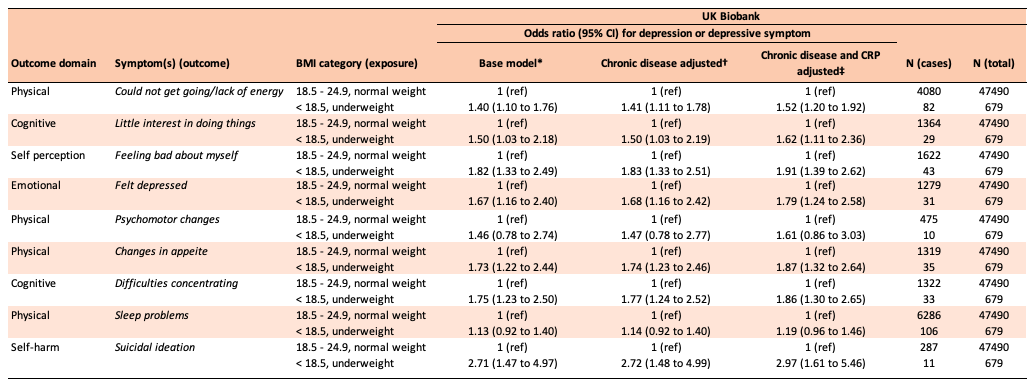


*Odds ratios adjusted for age and sex.

†Odds ratios adjusted for age, sex, chronic illnesses (i.e., vascular/ heart diseases, cancer, diabetes)

‡Odds ratios adjusted for age, sex, chronic illnesses (i.e., vascular/ heart diseases, cancer, diabetes), and C-reactive protein.

Appendix 2. Study specific estimates for the association between body mass index and individual depressive symptoms

FIGURE S11a. Forest plot for the cross-sectional association between overweight and individual depression symptoms (adjusted for age & sex)


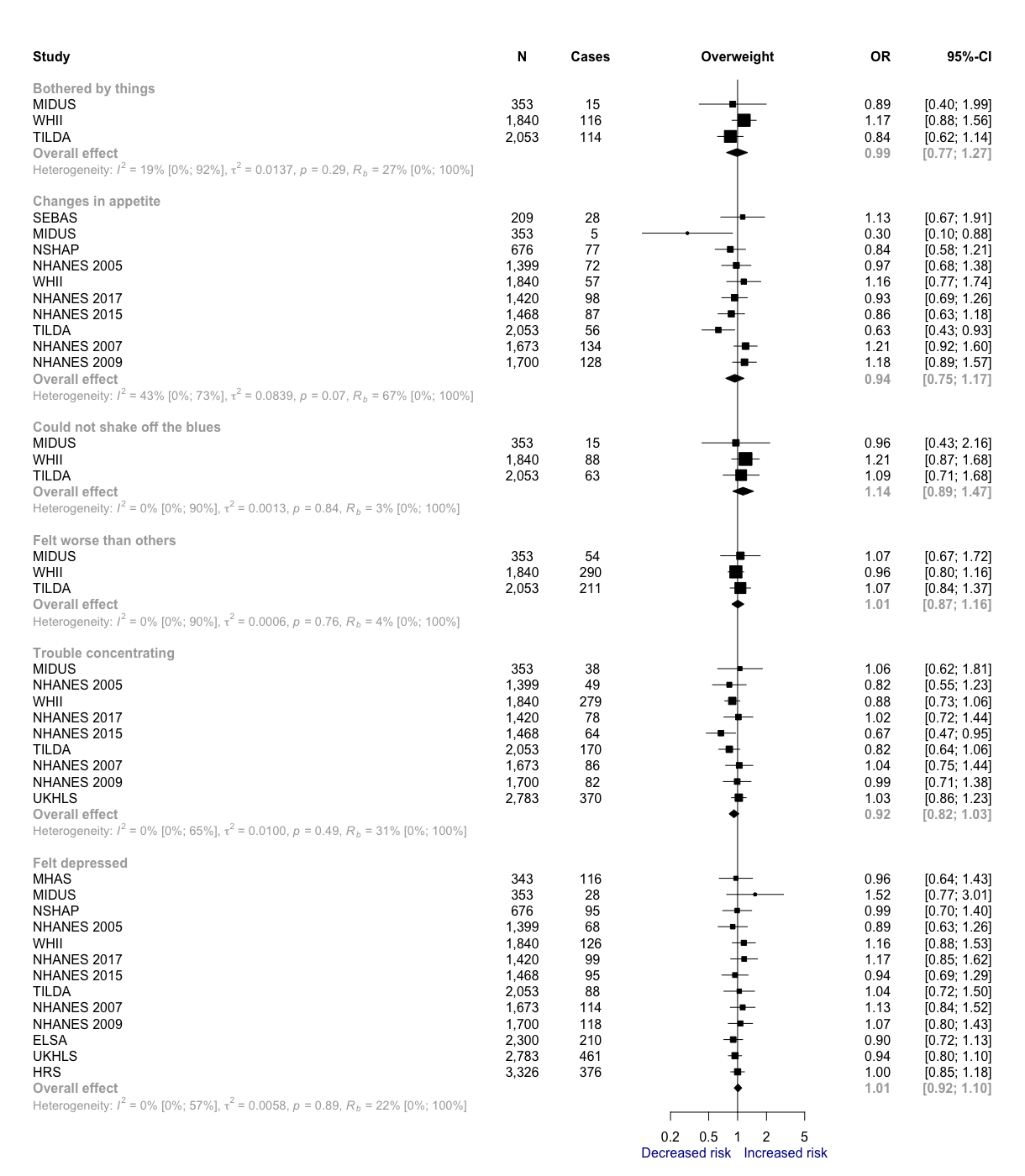


***Figure S11a* *(continued)***


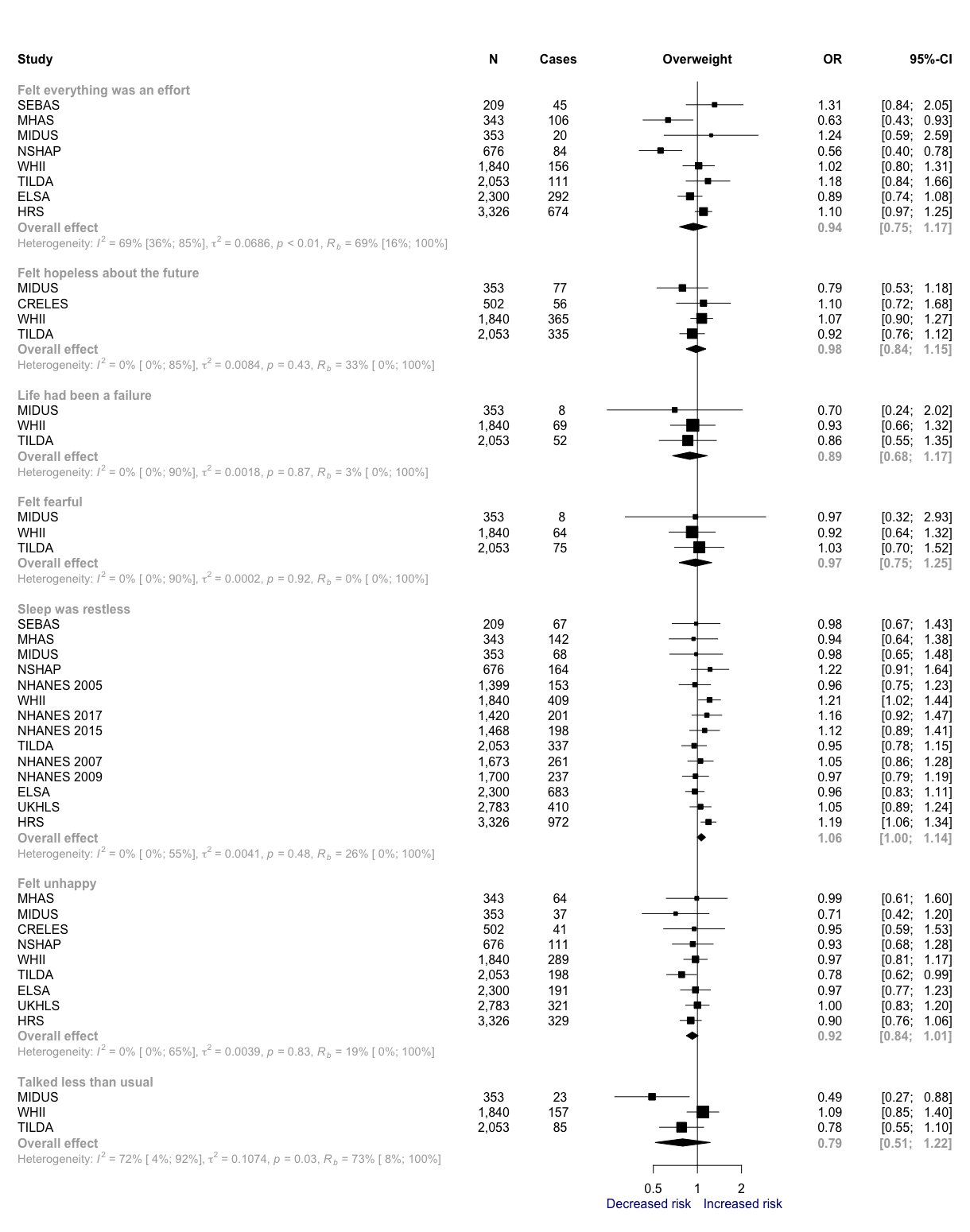


***Figure S11a* *(continued)***

*
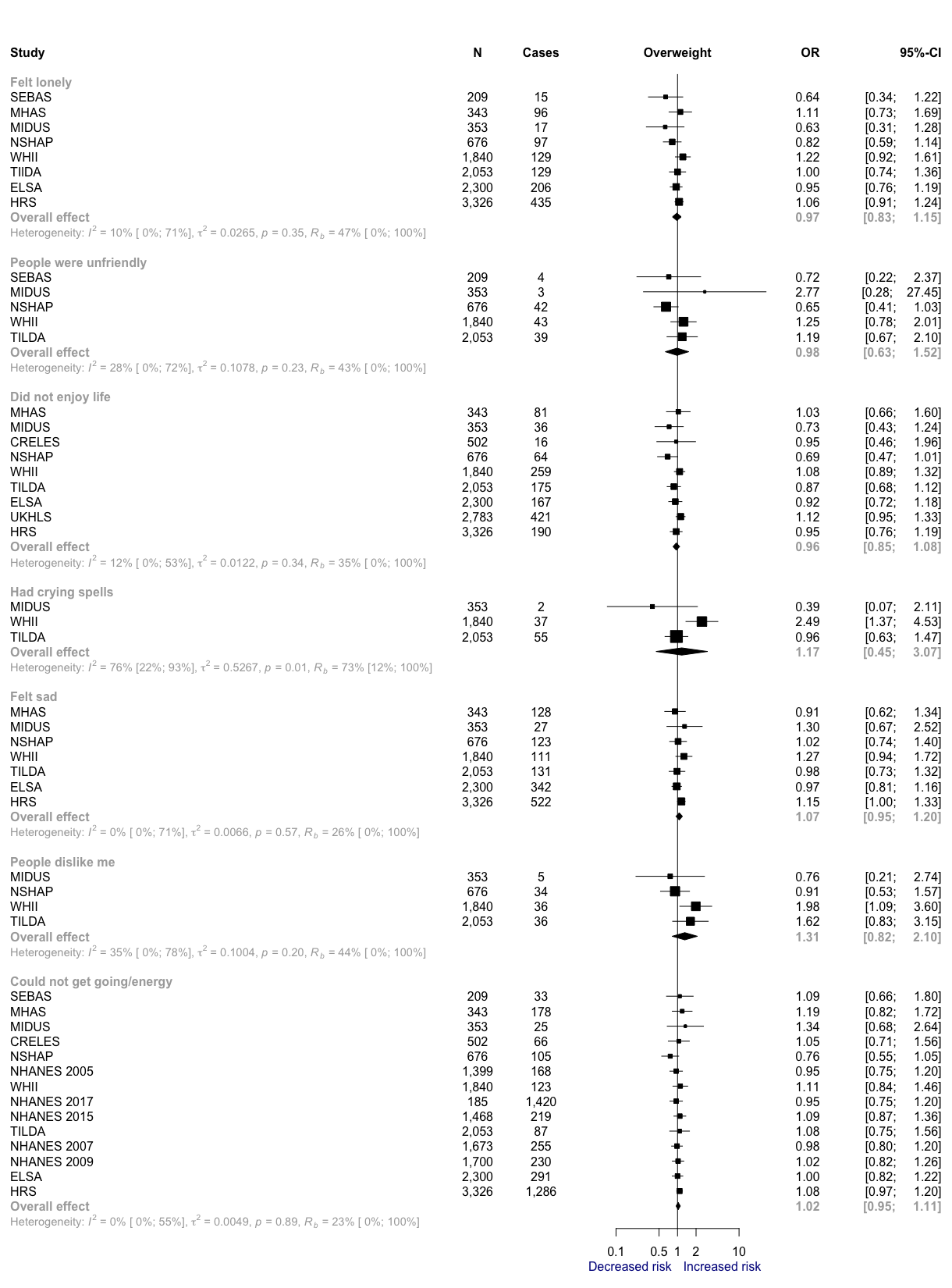
*

***Figure S11* *(continued)***

**
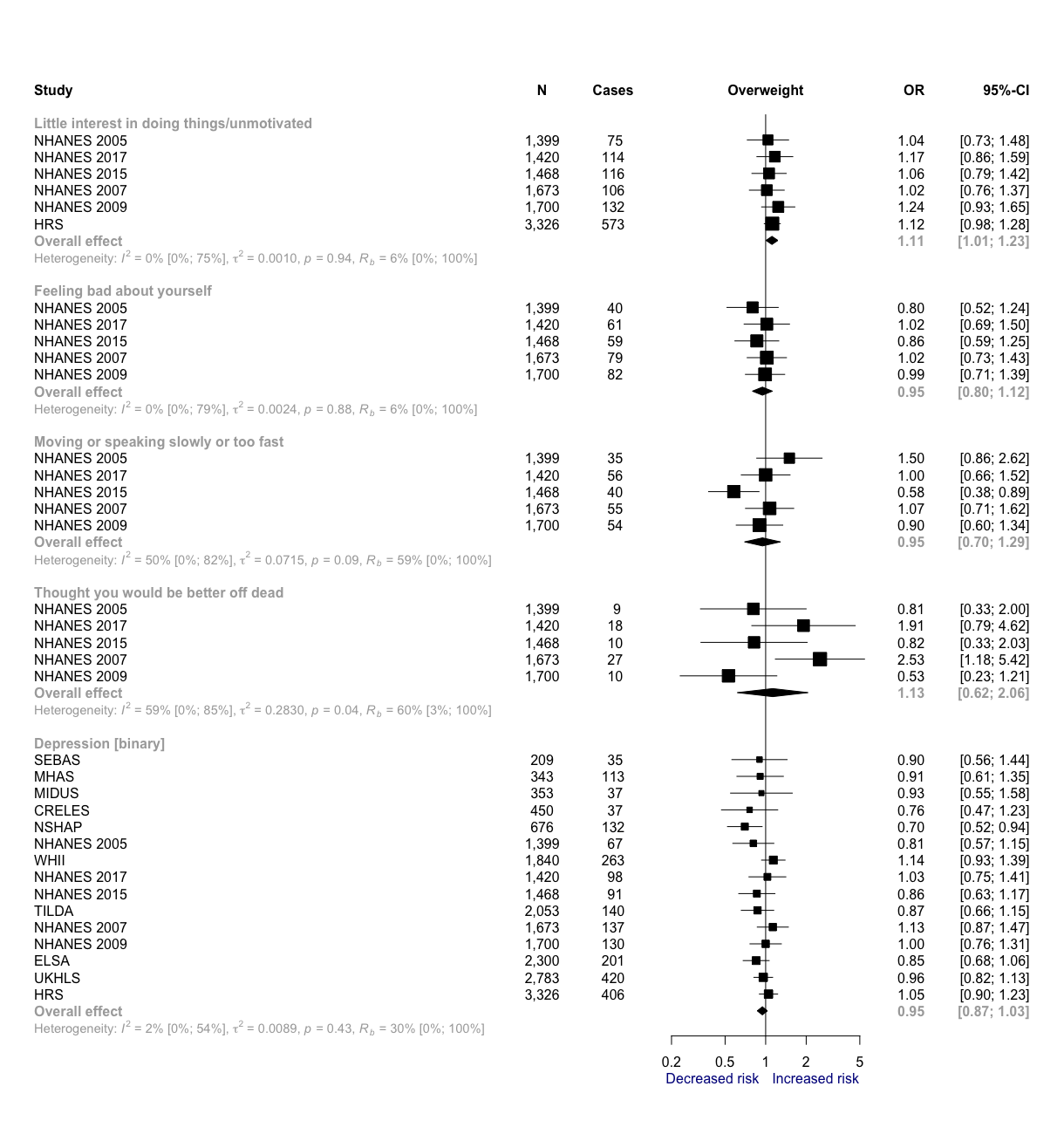
**

FIGURE S11b. Forest plot for the cross-sectional association between obesity class I and individual depression symptoms (adjusted for age & sex)

**
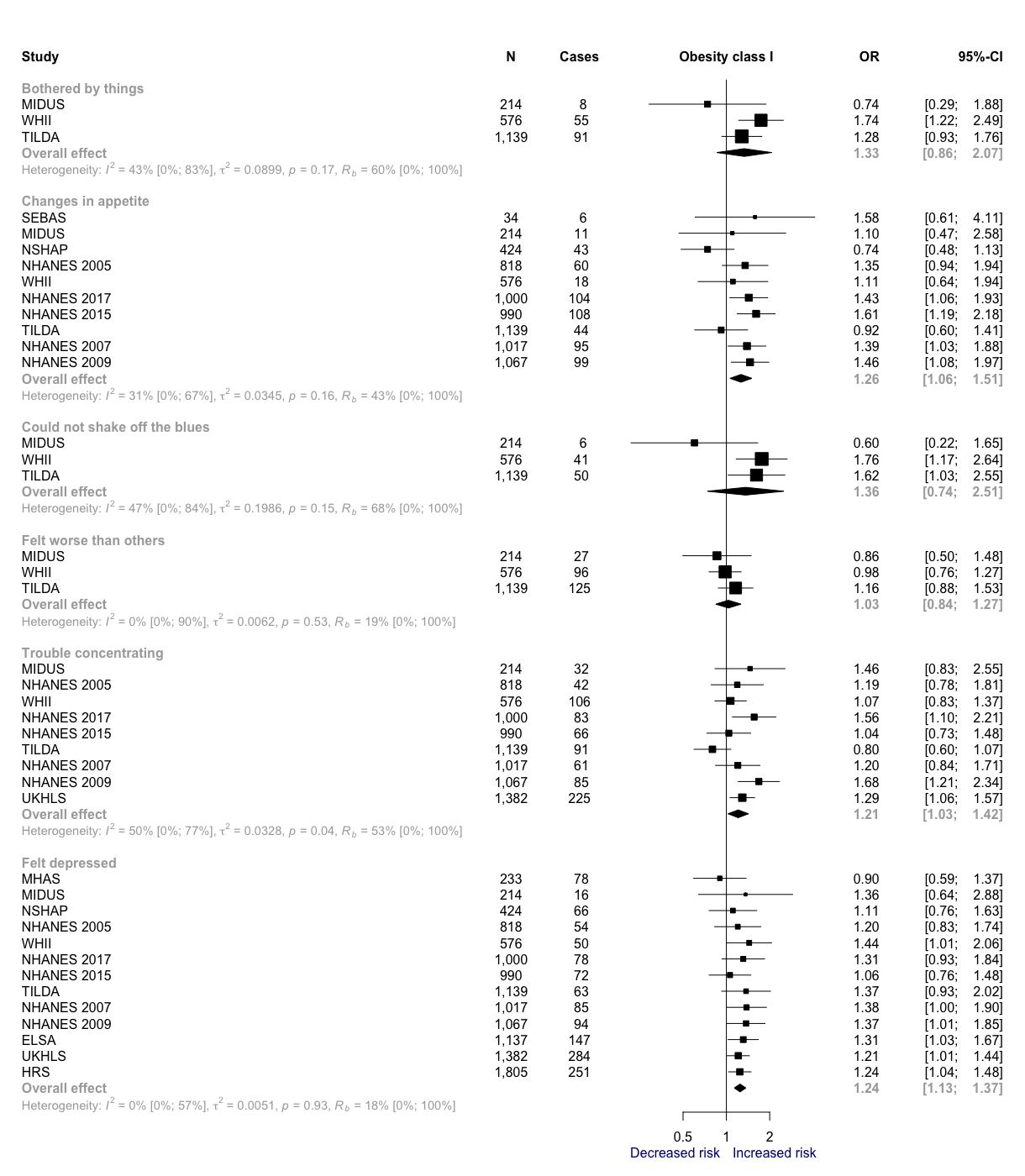
**

***Figure S11b* *(continued)***

**
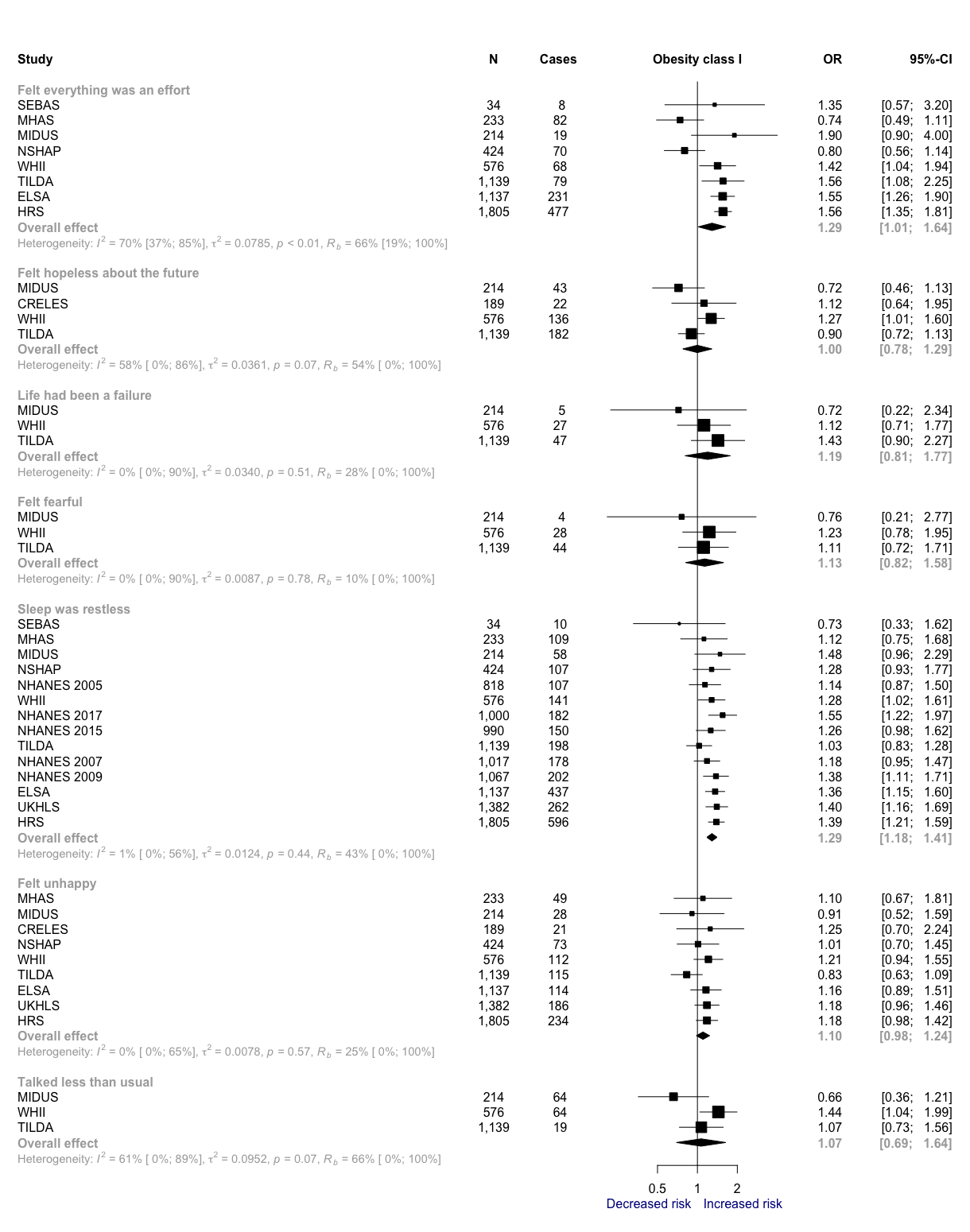
**

***Figure S11b* *(continued)***

**
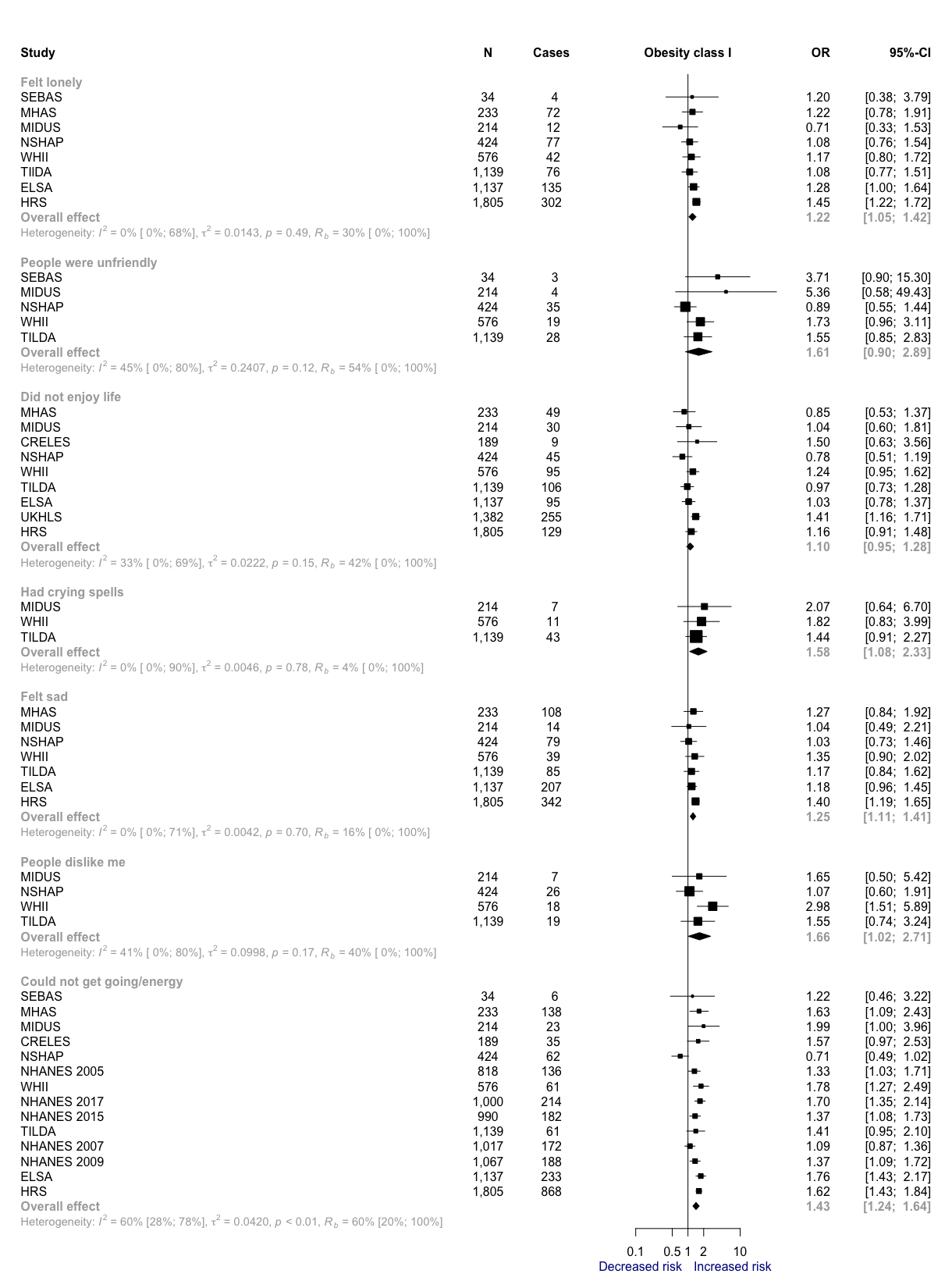
**

***Figure S11b* *(continued)***


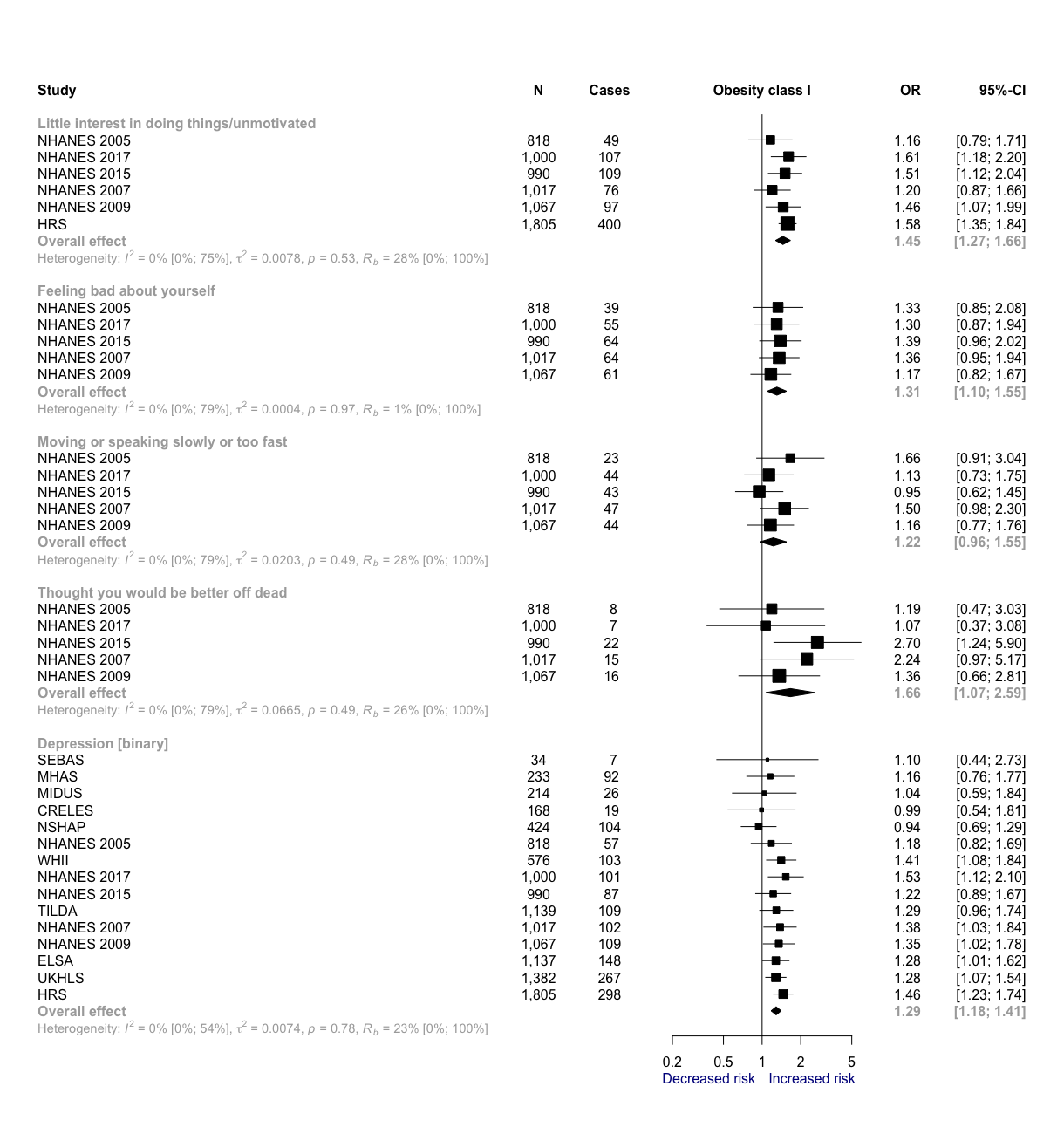


FIGURE S11c. Forest plot for the cross-sectional association between obesity class II and individual depression symptoms (adjusted for age & sex)


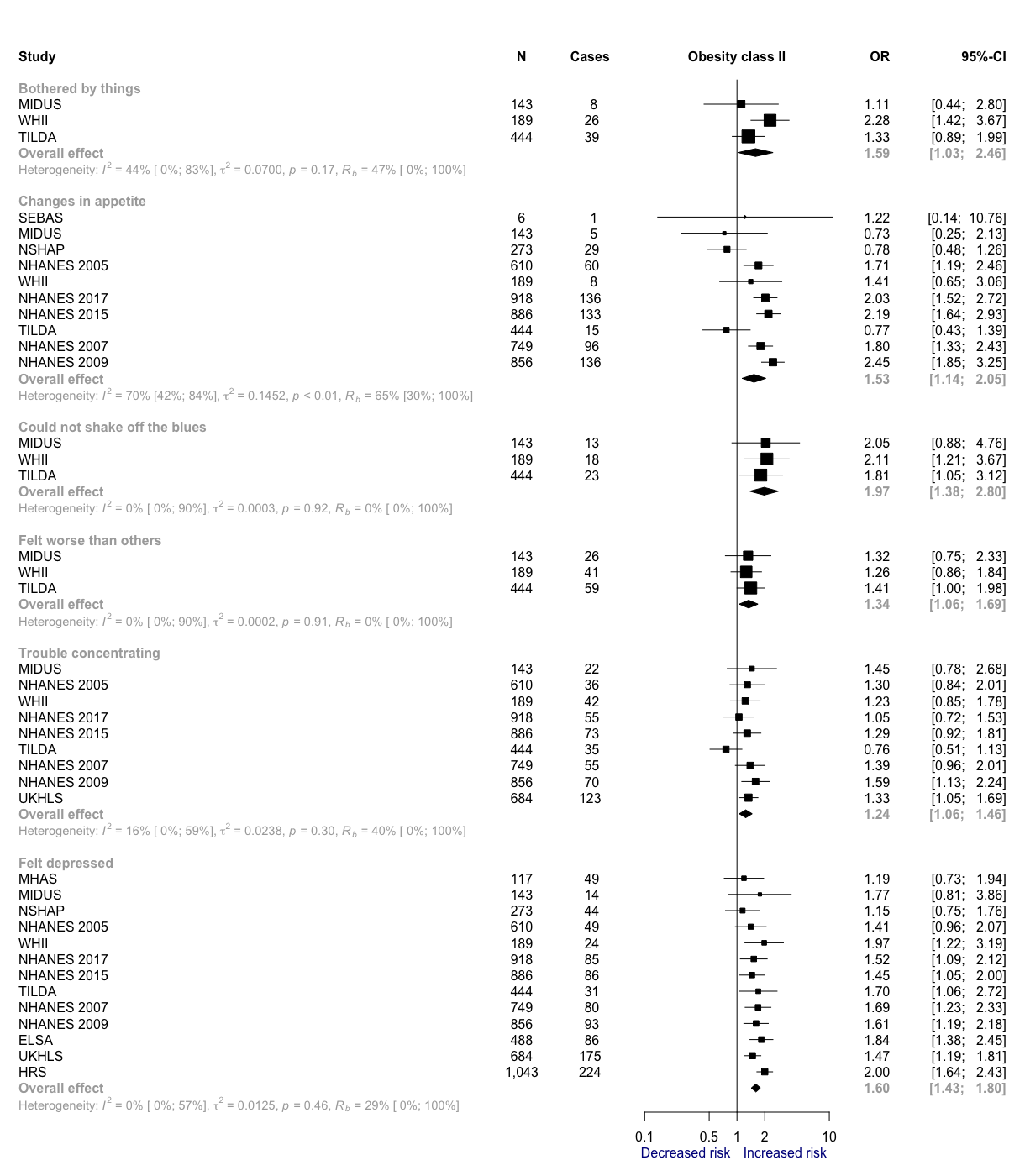


***Figure S11c* *(continued)***

*
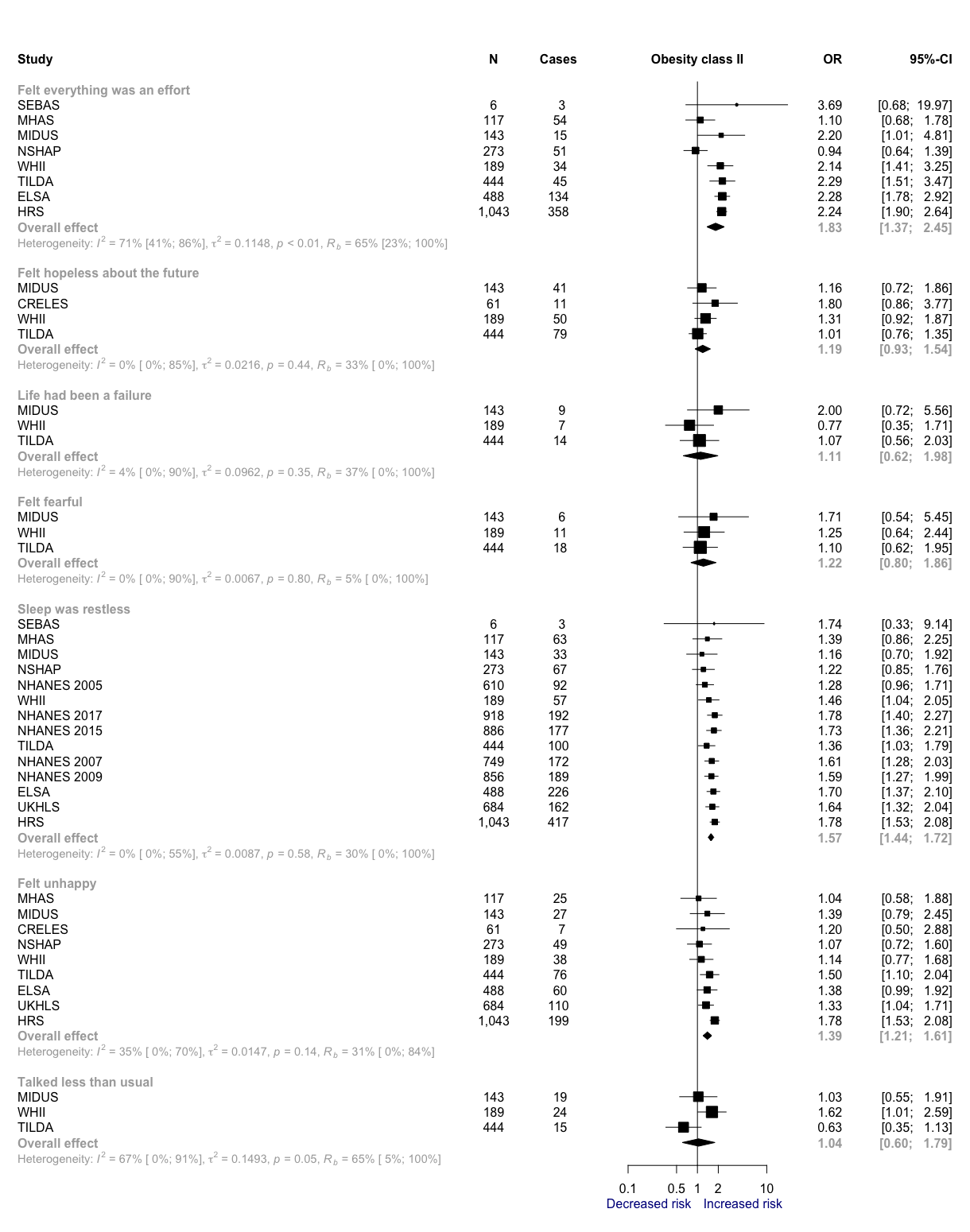
*

***Figure S11c* *(continued)***


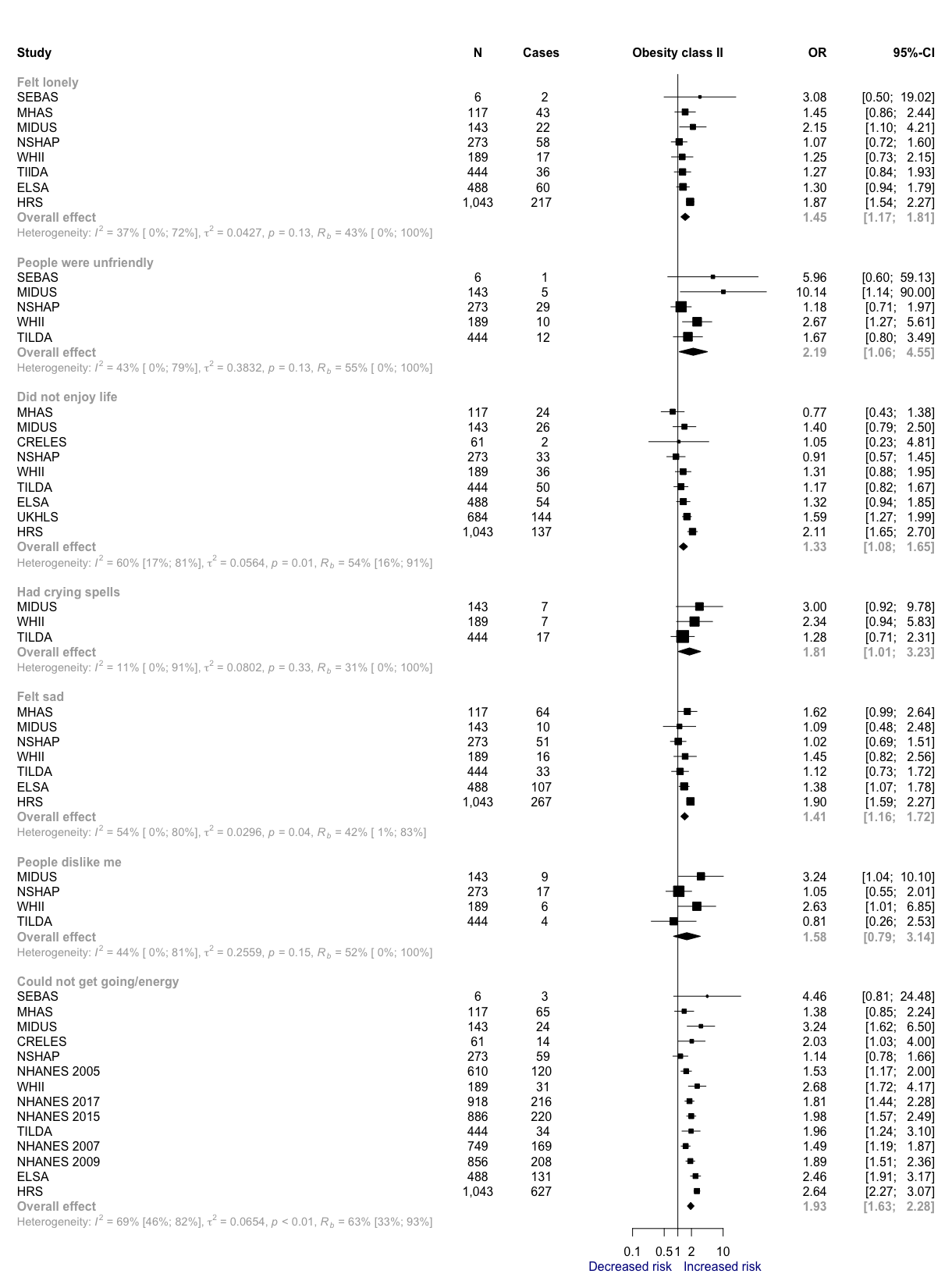


***Figure S11c* *(continued)***


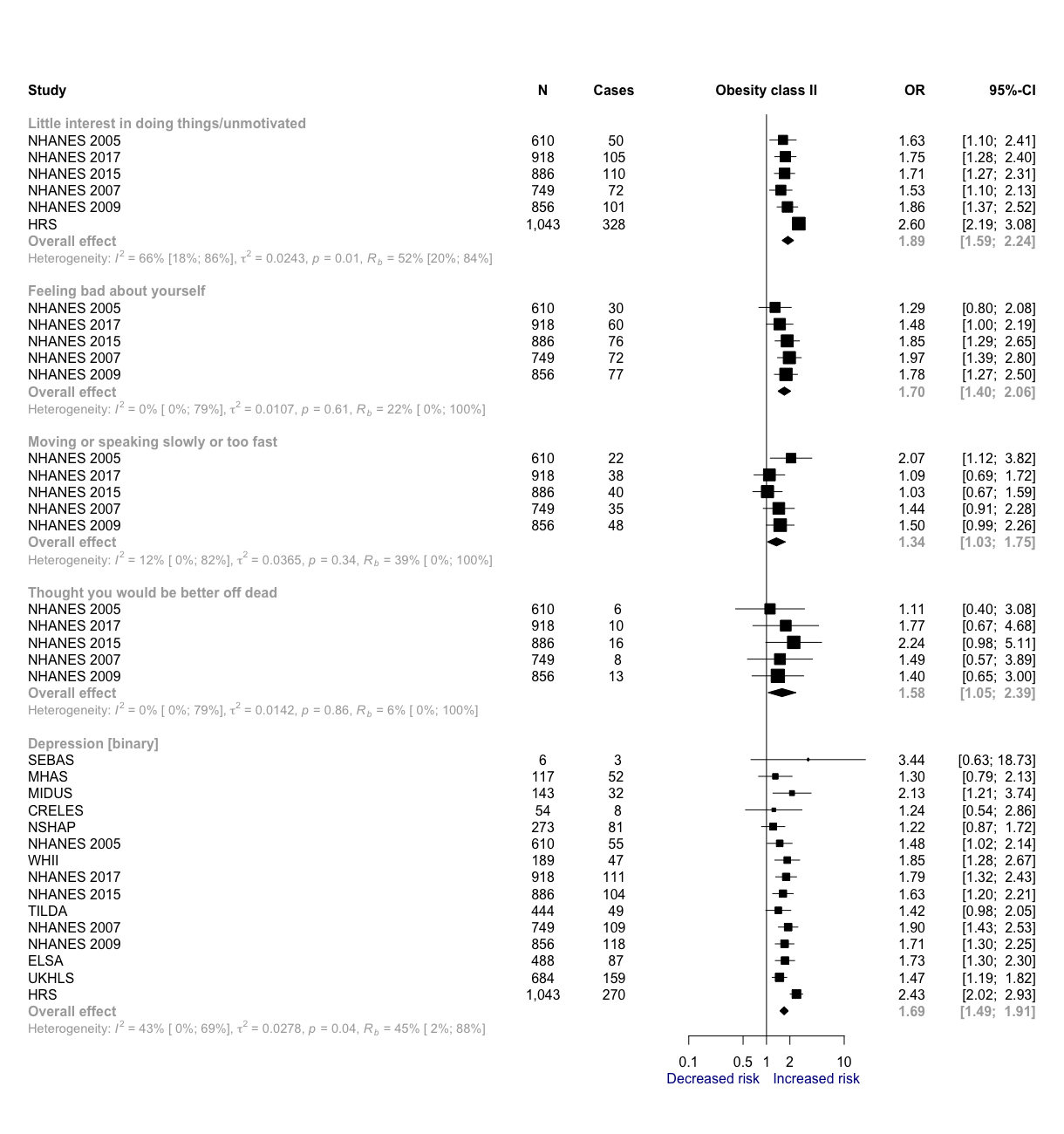


FIGURE S12a. Forest plot for the longitudinal association between overweight and individual depression symptoms at follow-up (adjusted for age, sex, and symptom at baseline)


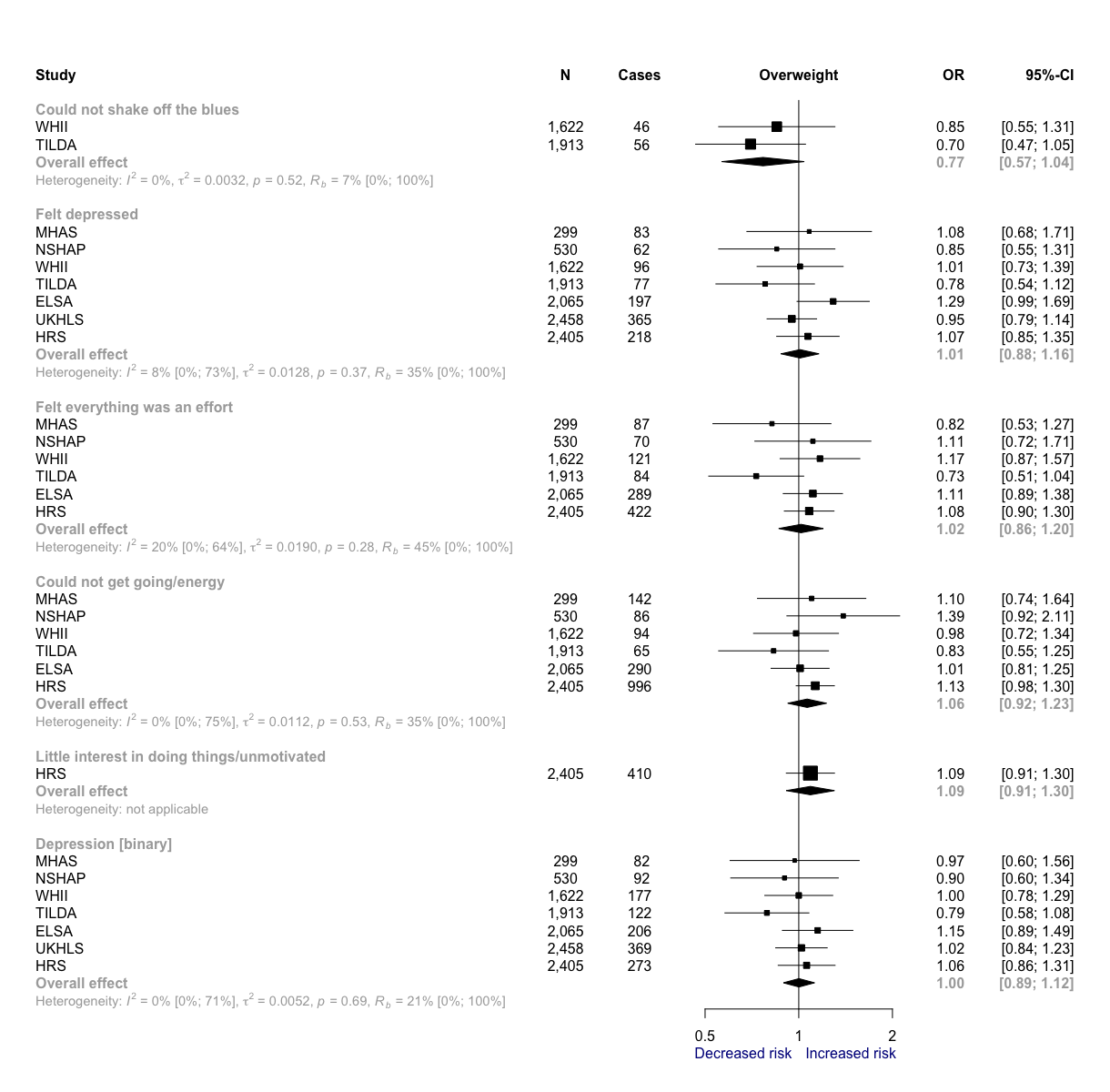


FIGURE S12b. Forest plot for the longitudinal association between obesity class I and individual depression symptoms at follow-up (adjusted for age, sex, and symptom at baseline)


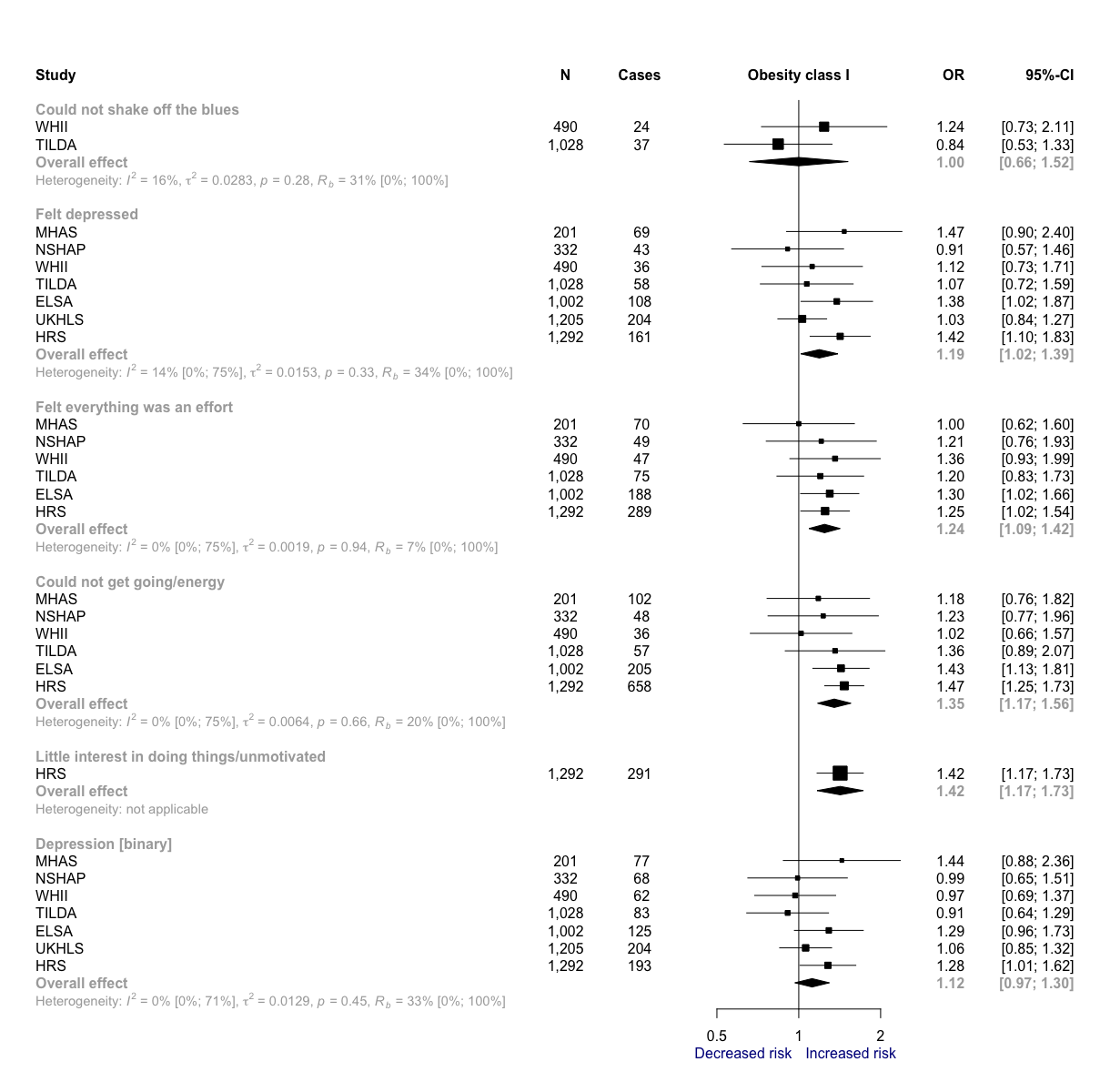


FIGURE S12c. Forest plot for the longitudinal association between obesity class II and individual depression symptoms at follow-up (adjusted for age, sex, and symptom at baseline)


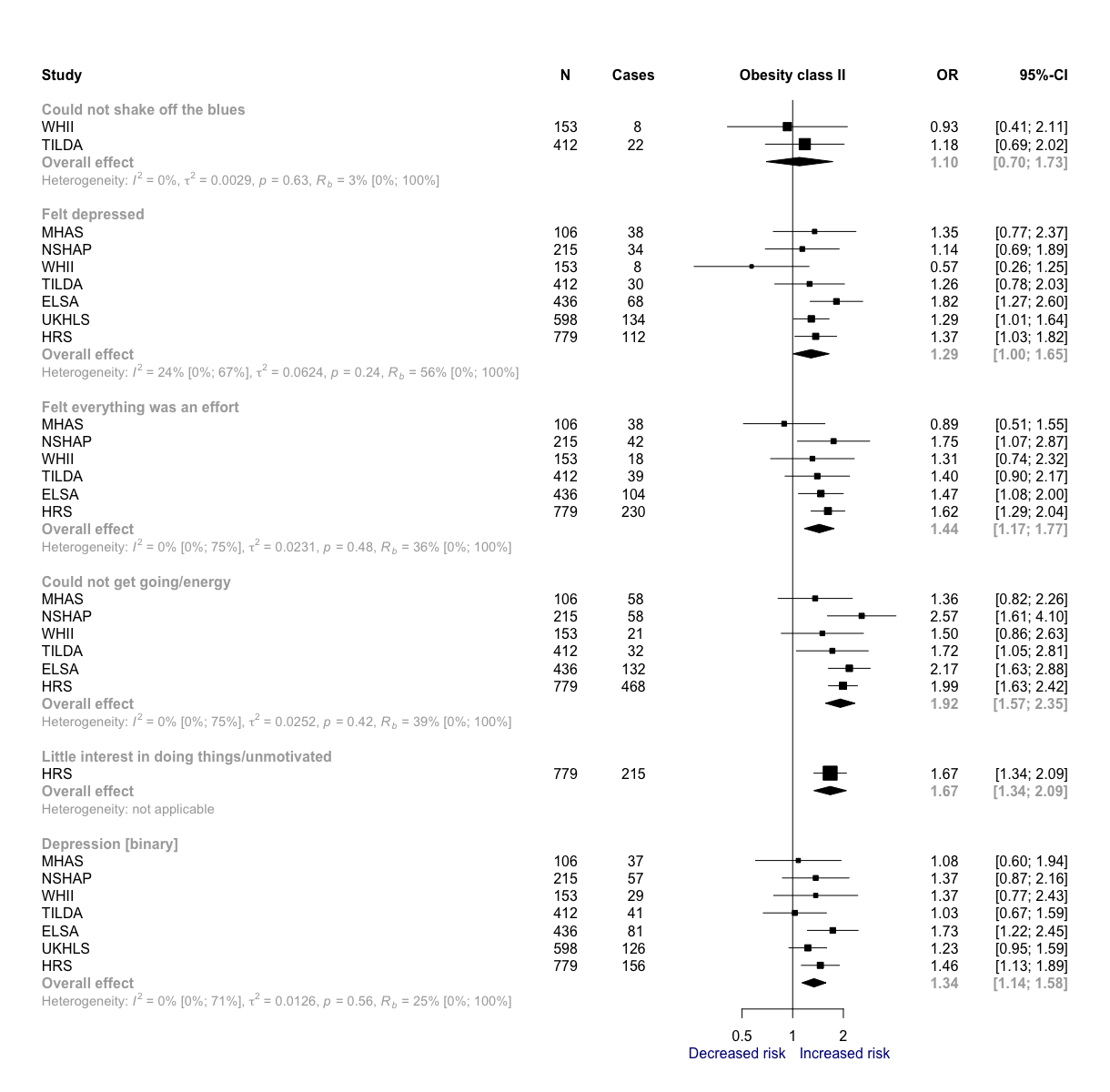


Appendix 3. Statistical Code

Statistical Code R: Random-effects meta-analysis of the cross-sectional associations between body mass index categories and individual symptoms of depression (RStudio)


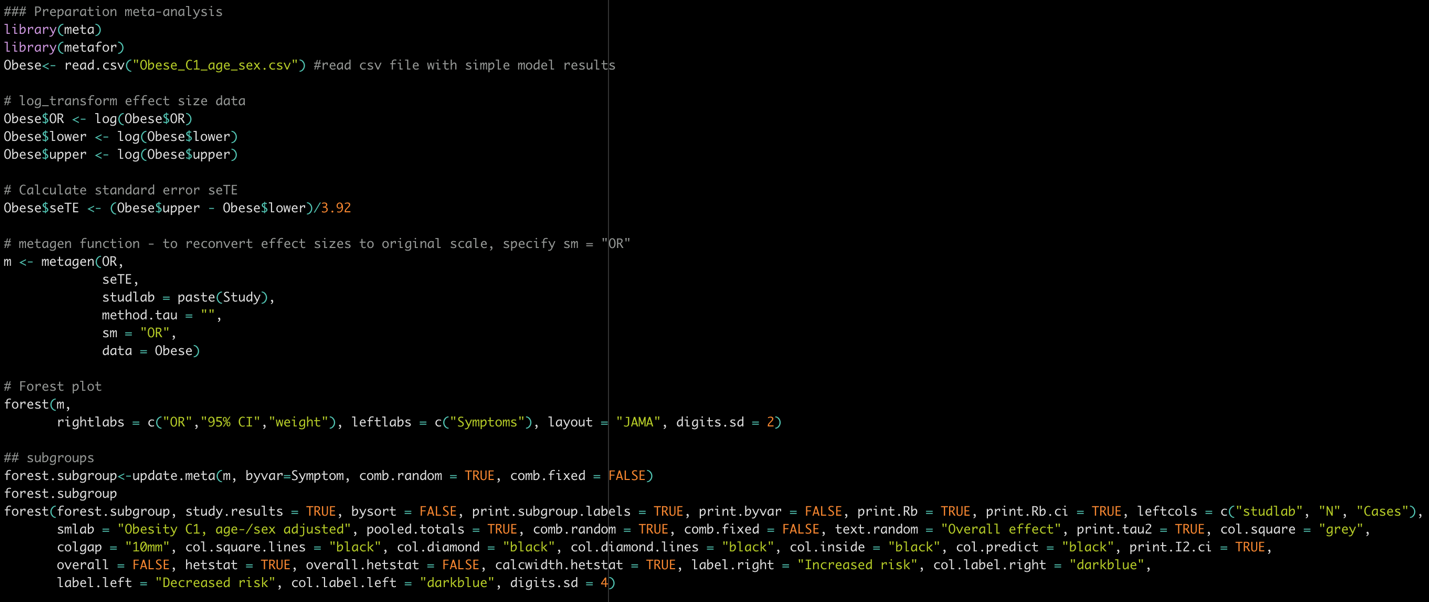


Statistical Code STATA: Loop for multivariate logistic regression analyses (example)

The following code is an example syntax (i.e., loop) used to calculate the odds ratios and accompanying 95% confidence intervals for the cross-sectional association between body mass index categories and individual depression symptoms in Whitehall II.


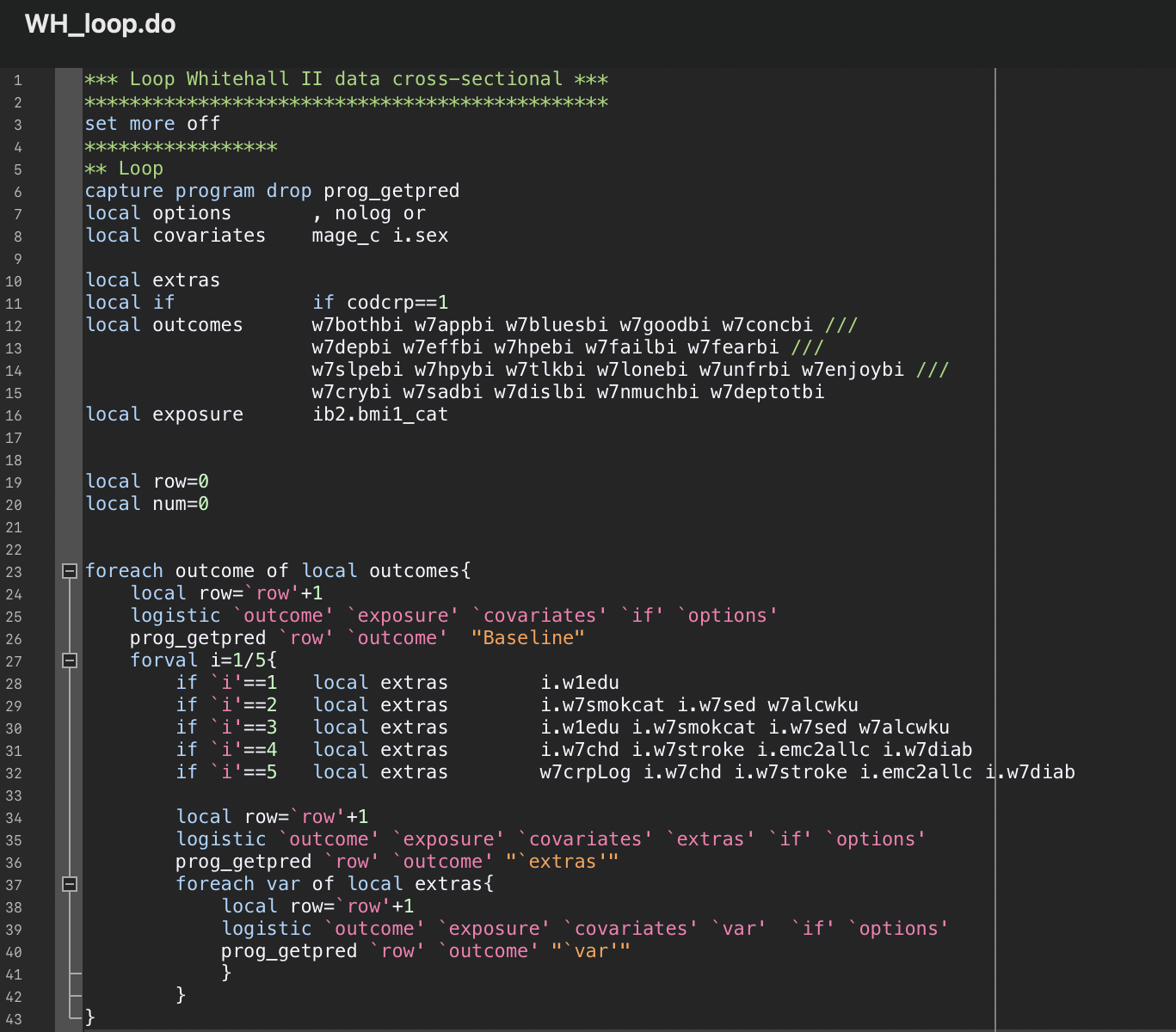


References

1. Rosero-Bixby L. CRELES: Costa Rican Longevity and Healthy Aging Study, 2005 (Costa Rica Estudio de Longevidad y Envejecimiento Saludable); 2010.

2. Sheikh JI, Yesavage JA, Brooks JO, et al. Proposed factor structure of the Geriatric Depression Scale. *International Psychogeriatrics* 1991; **3**(1): 23-8.

3. Steptoe A, Breeze E, Banks J, Nazroo J. Cohort profile: the English longitudinal study of ageing. *International journal of epidemiology* 2013; **42**(6): 1640-8.

4. Radloff LS. The CES-D scale: A self-report depression scale for research in the general population. *Applied psychological measurement* 1977; **1**(3): 385-401.

5. Ryff CD, Seeman T, Weinstein M. National Survey of Midlife Development in the United States (MIDUS II): Biomarker Project, 2004-2009: Inter-university Consortium for Political and Social Research; 2010.

6. Marmot M, Brunner E. Cohort profile: the Whitehall II study. *International journal of epidemiology* 2005; **34**(2): 251-6.

7. Buck N, McFall S. Understanding Society: design overview. *Longitudinal and Life Course Studies* 2011; **3**(1): 5-17.

8. Goldberg DP. The detection of psychiatric illness by questionnaire. *Maudsley monograph* 1972; **21**.

9. Centers for Disease Control Prevention. National Center for Health Statistics (NCHS). National Health and Nutrition Examination Survey Questionnaire (or Examination Protocol, or Laboratory Protocol). [*http://www*](http://www) *cdc gov/nchs/nhanes htm* 2006.

10. Kroenke K, Spitzer RL, Williams JB. The PHQ‐9: validity of a brief depression severity measure. *Journal of general internal medicine* 2001; **16**(9): 606-13.

11. Cornman JC, Glei DA, Goldman N, et al. Cohort profile: the social environment and biomarkers of aging study (SEBAS) in Taiwan. *International journal of epidemiology* 2016; **45**(1): 54-63.

12. Heeringa SG, Connor JH. Technical description of the Health and Retirement Survey sample design. *Ann Arbor: University of Michigan* 1995.

13. Kearney PM, Cronin H, O'Regan C, et al. Cohort profile: the Irish longitudinal study on ageing. *International journal of epidemiology* 2011; **40**(4): 877-84.

14. O’Muircheartaigh C, English N, Pedlow S, Kwok PK. Sample design, sample augmentation, and estimation for Wave 2 of the NSHAP. *Journals of Gerontology Series B: Psychological Sciences and Social Sciences* 2014; **69**(Suppl_2): S15-S26.

15. Wong R, Michaels-Obregon A, Palloni A. Cohort profile: the Mexican health and aging study (MHAS). *International journal of epidemiology* 2017; **46**(2): e2-e.
